# Genetic inactivation of the Carnitine/Acetyl-Carnitine mitochondrial carrier of *Yarrowia lipolytica* leads to enhanced odd-chain fatty acid production

**DOI:** 10.1186/s12934-023-02137-8

**Published:** 2023-07-13

**Authors:** Eugenia Messina, Camilla Pires de Souza, Claudia Cappella, Simona Nicole Barile, Pasquale Scarcia, Isabella Pisano, Luigi Palmieri, Jean-Marc Nicaud, Gennaro Agrimi

**Affiliations:** 1 Department of Biosciences, Biotechnology and Environment, University of Bari “Aldo Moro”, Campus Universitario, via Orabona 4, Bari, 70125 Italy; 2 Université Paris-Saclay, INRAE, Micalis Institute, Jouy-en-Josas, 78350 AgroParisTech France; 3 Bioenergetics and Molecular Biotechnologies (IBIOM), CNR Institute of Biomembranes, Campus Universitario, via Orabona 4, Bari, 70125 Italy

**Keywords:** *Yarrowia lipolytica*, Mitochondrial carrier, Carnitine/Acetyl-Carnitine shuttle, Acetyl-CoA, Metabolic engineering, Odd-chain fatty acids (OCFAs)

## Abstract

**Background:**

Mitochondrial carriers (MCs) can deeply affect the intracellular flux distribution of metabolic pathways. The manipulation of their expression level, to redirect the flux toward the production of a molecule of interest, is an attractive target for the metabolic engineering of eukaryotic microorganisms. The non-conventional yeast *Yarrowia lipolytica* is able to use a wide range of substrates. As oleaginous yeast, it directs most of the acetyl-CoA therefrom generated towards the synthesis of lipids, which occurs in the cytoplasm. Among them, the odd-chain fatty acids (OCFAs) are promising microbial-based compounds with several applications in the medical, cosmetic, chemical and agricultural industries.

**Results:**

In this study, we have identified the MC involved in the Carnitine/Acetyl-Carnitine shuttle in *Y. lipolytica*, YlCrc1. The *Y. lipolytica* Yl*crc1* knock-out strain failed to grow on ethanol, acetate and oleic acid, demonstrating the fundamental role of this MC in the transport of acetyl-CoA from peroxisomes and cytoplasm into mitochondria. A metabolic engineering strategy involving the deletion of Yl*CRC1*, and the recombinant expression of propionyl-CoA transferase from *Ralstonia eutropha* (RePCT), improved propionate utilization and its conversion into OCFAs. These genetic modifications and a lipogenic medium supplemented with glucose and propionate as the sole carbon sources, led to enhanced accumulation of OCFAs in *Y. lipolytica*.

**Conclusions:**

The Carnitine/Acetyl-Carnitine shuttle of *Y. lipolytica* involving YlCrc1, is the sole pathway for transporting peroxisomal or cytosolic acetyl-CoA to mitochondria. Manipulation of this carrier can be a promising target for metabolic engineering approaches involving cytosolic acetyl-CoA, as demonstrated by the effect of Yl*CRC1* deletion on OCFAs synthesis.

**Supplementary Information:**

The online version contains supplementary material available at 10.1186/s12934-023-02137-8.

## Background

The ability to use a wide range of carbon sources including hydrophobic molecules, its Generally Recognized As Safe (GRAS) status, the available effective tools for its genetic manipulation and its capacity to accumulate lipids to over 20% of its biomass, make the non-conventional yeast *Yarrowia lipolytica* an increasingly important industrial host for biotechnological applications [1–4]. A key metabolic trait that contributes to the oleaginous phenotype of this yeast is its very active carbon flux toward acetyl-CoA formation and consequently, the high potential for lipid and lipid-like molecules synthesis [5, 6]. When carbohydrates are used as carbon sources, acetyl-CoA is synthesized in the mitochondrial matrix, via the pyruvate dehydrogenase complex (*PDH*), and import of acetyl-CoA across the mitochondrial membrane is not required. *Y. lipolytica* can efficiently use as carbon and energy source, substrates leading to acetyl-CoA formation in the cytosol or in peroxisomes such as, ethanol, acetate and fatty acids (FAs). The cytosolic acetyl-CoA synthetase gene, *ACS1* (YALI0F05962g), is required for the metabolism of ethanol and acetic acid [7]. The catabolism of FAs takes place in peroxisomes via the β-oxidation pathway, a four-reaction cyclic pathway that shortens the FA backbone releasing acetyl-CoA [8]. In order to enter the TCA cycle, the acetyl-CoA derived from the abovementioned pathways has to be transported across the inner mitochondrial membrane, which is impermeable to this compound. Mitochondrial carries (MCs) are responsible for the translocation of hydrophilic metabolites, such as inorganic anions, di- and tri-carboxylates, keto acids, amino acids, nucleotides and coenzymes, across the mitochondrial and the peroxisomal membranes. The protein sequences of MCs are characterized by three tandemly repeated domains of about 100 residues, containing two hydrophobic segments and a characteristic signature sequence motif [9]. The Carnitine/Acyl-Carnitine (CAC) mitochondrial carrier is a conserved protein among different organisms, and it catalyzes the exchange of carnitine and acyl-carnitines with carbon chain length from 2 to 18 carbon atoms, through the inner mitochondrial membrane. The mammalian CAC has a higher affinity for long chain acyl-carnitines, whereas the fungal transporters for short chain acyl-carnitines [10]. Among yeasts, only the *S. cerevisiae* CAC mitochondrial carrier (ScCrc1) has been reconstituted in liposomes and biochemically characterized, in terms of substrate specificity: it transports carnitine, acetyl-carnitine, propionyl-carnitine and to a much lower extent medium- and long-chain acyl-carnitines [11]. The acetyl-CoA transport process between cellular compartments in *Y. lipolytica* is surprisingly poorly studied. Cytosolic acetyl-CoA is a key precursor of several biotechnologically important products such as isoprenoids, long-chain alcohols, sterols, polyketides, polyphenols and fatty acids [12]. In *Y. lipolytica de novo* FAs synthesis is triggered when a non-carbon nutrient, such as nitrogen, becomes growth limiting and occurs in the cytosol starting from acetyl-CoA. The production of specialized lipid products can be obtained via fermentation and biocatalysis using low-cost carbon substrates and producing value-added molecules, not obtainable via traditional petrochemical processes, such as odd-chain fatty acids (OCFAs) [13, 14]. OCFAs represent less than 3% of the total FAs naturally synthetized by microorganisms. They have several important industrial applications being employed in the synthesis of pesticides, flavour and fragrance compounds, hydraulic fluids, plasticizers and coatings [15, 16]. Furthermore, the *cis*-9-heptadecenoic acid (C17:1) is employed in the pharmaceutical industry due to its anti-inflammatory effect and it is used to treat psoriasis, allergies and autoimmune diseases [17].Pentadecanoic Acid (C15:0) and heptadecanoic acid (C17:0) are predictor biomarkers for coronary heart disease (CHD), type II diabetes (T2D) obesity, and dairy fat intake during dietary assessment [18]. A crucial step in the synthesis of OCFAs is the production of their biosynthetic precursor, propionyl-CoA, which replaces acetyl-CoA in the first condensation step of FAs synthesis. The condensation of propionyl-CoA and malonyl-CoA produces the 3-oxovaleryl-ACP; this five-carbon compound then goes through a chain elongation process, where two carbons are added in each cycle. In *Y. lipolytica*, exogenous propionate (C3) has been used as a primer for the synthesis of OCFAs [13, 19] after its conversion into propionyl-CoA. Propionate is a volatile fatty acid (VFA) that can be obtained from anaerobic digestion of organic wastes. *Y. lipolytica* can readily use VFAs as feedstock for lipid production and thus can represents a promising cell factory for upgrading organic wastes [20]. In addition to propionate supplementation, the heterologous expression of propionyl-CoA transferase enzyme of *Ralstonia eutropha* (RePCT) in *Y. lipolytica*, has been shown to further increase the precursor pool for OCFAs synthesis, improving the accumulation of these specialized lipid products [19]. In this study, the Carnitine/Acetyl-Carnitine mitochondrial carrier of the oleaginous yeast *Y. lipolytica* has been identified, and its role in the acetyl-CoA shuttle from cytosol and peroxisomes to mitochondria has been assessed. The biochemical knowledge gained about acetyl-CoA inter-compartmental transport in *Y. lipolytica* has been used to improve OCFAs production. This is the first time that a metabolic engineering approach, involving the manipulation of the expression level of a MC, has been used to increase the synthesis of specialized lipids in *Y. lipolytica*.

## Results

### Identification of Carnitine/Acetyl-Carnitine transporter in Yarrowia lipolytica

**Fig. 1:**
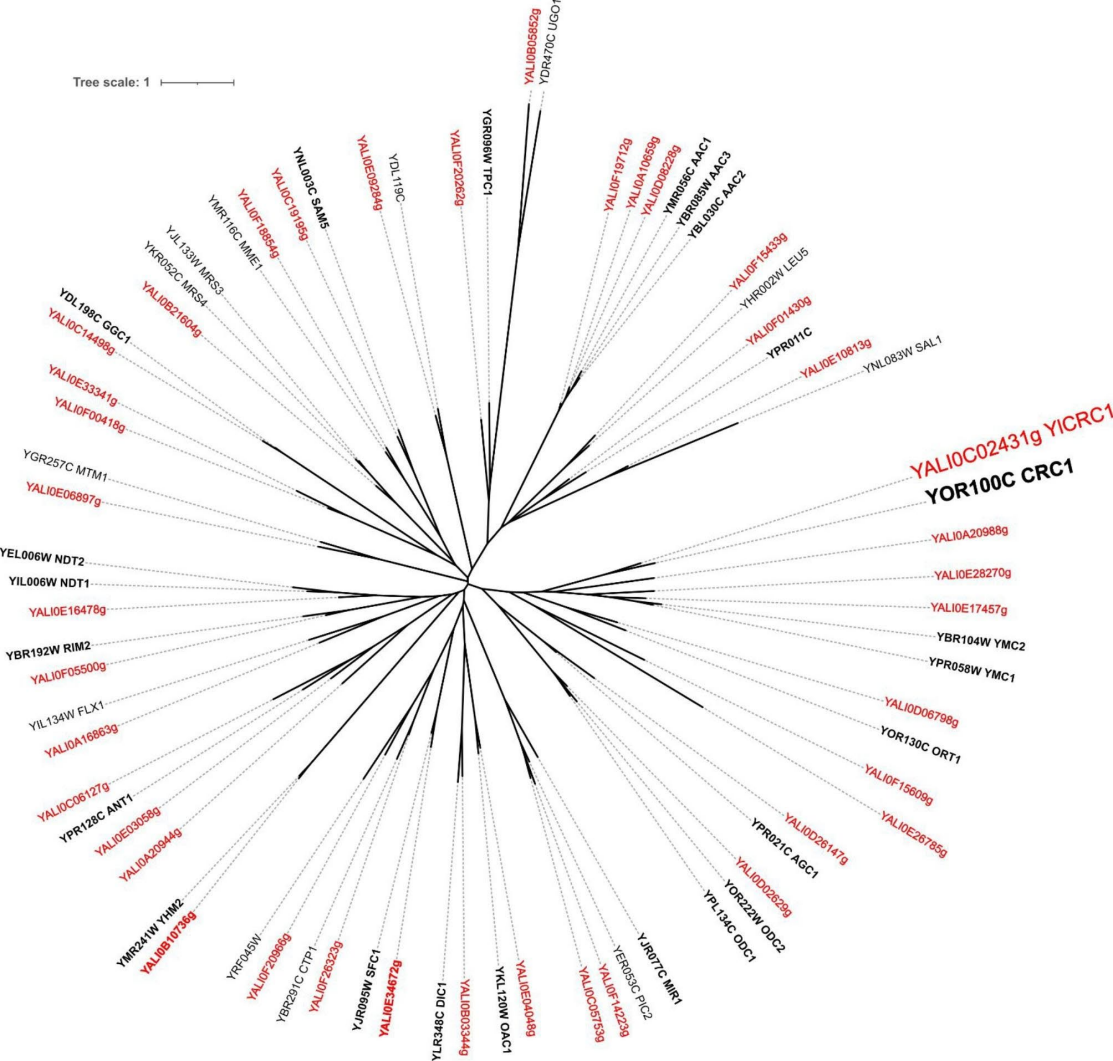
Phylogenetic tree of mitochondrial carriers of *Saccharomyces cerevisiae* and *Yarrowia lipolytica*. The tree was constructed using the sequences containing the signature motifs characterizing the mitochondrial carrier family (PFAM number PF00153) from *S. cerevisiae* (black) and *Y. lipolytica* (red). The names of the mitochondrial carriers and/or their coding genes are found on the terminal nodes

*Y. lipolytica* protein database was screened for sequences containing the signature motifs characterizing the mitochondrial carrier family (PFAM number PF00153), obtaining 39 sequences. A phylogenetic tree (Fig. 1) was built using these sequences along with those of *S. cerevisiae* mitochondrial carriers for many of which, a function has been assigned (names in bold in Fig. 1) [21].

**Fig. 2:**
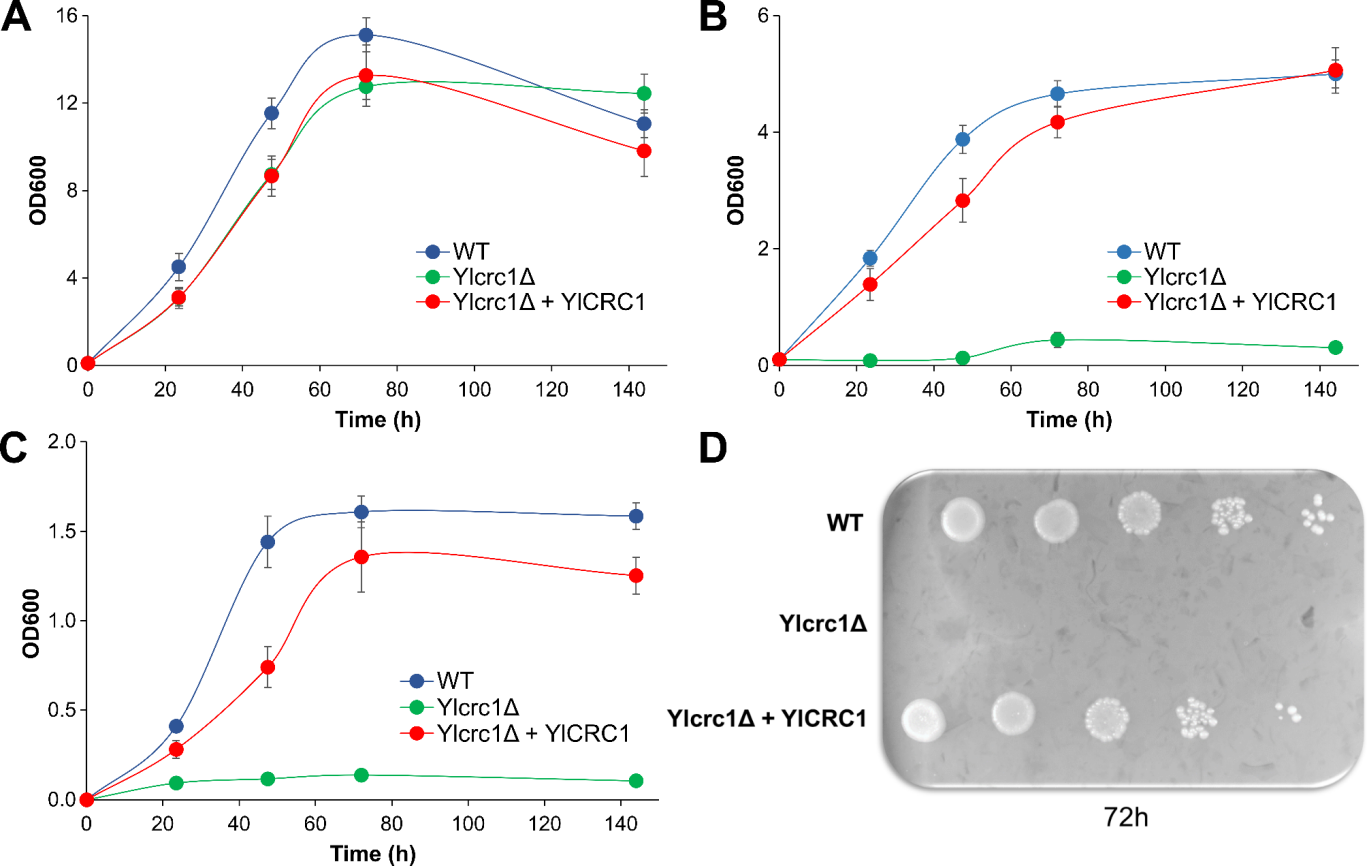
Growth of wild type (blue), strain with deletion of Yl*CRC1* (green) and Yl*crc1*Δ + Yl*CRC1* strain (red) on YNB medium with different carbon sources (20 g/L) **A**) glucose, **B**) acetate, and **C**) ethanol. Averages and standard errors were obtained from at least three replicate experiments. **D**) Comparison of growth in wild type, Yl*crc1*Δ strain and Yl*crc1*Δ + Yl*CRC1* strain on solid YNB medium with 10 g/L oleic acid, at 72 h of incubation at 28 °C

The phylogenetic tree shows that for each *S. cerevisiae* MC, a *Y. lipolytica* putative ortholog can be identified. The *S. cerevisiae* CAC mitochondrial transporter, ScCrc1 [11], clusters with ScOrt1 and ScYmc1/2 identified as basic amino acids and glutamate transporters, respectively [22, 23]. The *Y. lipolytica* sequence closest to ScCrc1 appears to be the protein encoded by the YALI0C02431 gene. A more distant homolog of ScCrc1 is encoded by YALI0A20988g whereas the other two *Y. lipolytica* mitochondrial carriers of the clade appear to be more closely related to ScYmc1/2 (Fig. 1). The YALI0C02431 gene is found on chromosome C and encodes a protein of 314 amino acids that we named YlCrc1; YALI0A20988g is located on chromosome A and encodes a protein of 317 amino acids. The alignment of the amino acid sequences of ScCrc1, with those of YlCrc1 and YALI0A20988p (Additional Fig. 1) revealed an amino acids identity of 50% and 24.92% with YlCrc1 and YALI0A20988p, respectively (Additional Table 1). A microbial CAC transporter has been found also in *Aspergillus nidulans* [24] and shares 59.87% and 26.18% of amino acids identity with YlCrc1 and YALI0A20988p, respectively (Additional Table 1). The human CAC transporter [25] displays an amino acids identity of 37.87% with YlCrc1 and 28.23% with YALI0A20988p (Additional Table 1). Both YlCrc1 and YALI0A20988p display the characteristic signature motif of the mitochondrial carriers PX[DE]XX[RK]X[RK] [9] (Additional Fig. 1). YlCrc1 presents also a specific motif R-X-X-P-A-N-A-A-X-F [24] located at the sixth alpha-helix, which has been found in all the characterized CAC carriers (Additional Fig. 1). These amino acids, reported to be involved in substrate binding during Carnitine/Acetyl-Carnitine translocation or in conformational changes occurring during substrate translocation [26], are only partially conserved in YALI0A20988p (Additional Fig. 1) suggesting a different substate specificity. To shed light on the physiological function of YlCrc1, the deletion of its encoding gene was performed in *Y. lipolytica* via the dual cleavage CRISPR/Cas9 strategy [27]. In liquid glucose-containing minimal medium, the strain Yl*crc1*Δ exhibited growth characteristics similar to those of the wild type strain (Fig. 2A). In contrast, in liquid acetate and ethanol-containing minimal media the mutant strain failed to grow (Fig. 2B-C). In keeping with its putative role as a Carnitine/Acetyl-Carnitine carrier, the concentration of both substrates remained almost unchanged throughout the growth of Yl*crc1*Δ while, the wild type consumed about 15 g/L and 10 g/L of acetate and ethanol, respectively, in 72 h (Additional Fig. 2B-C). In addition, the deletion of Yl*CRC1* was lethal on solid minimal medium with oleic acid as sole carbon source (Fig. 2D). To check whether the differences in growth, displayed by the Yl*crc1*Δ strain, were the results of the absence of YlCrc1 and not a secondary effect, homologous expression of Yl*CRC1* gene in the Yl*crc1*Δ strain was evaluated. For this purpose, Yl*CRC1* was cloned under the strong constitutive translation elongation factor-1 promoter (pTEF). As shown in Fig. 2, the reintroduction of the gene completely restored the growth on acetate, ethanol and oleic acid.

All these data show that the function of YlCrc1 mitochondrial carrier is essential for *Y. lipolytica* growth on carbon sources generating acetyl-CoA in the cytosol or in peroxisomes. Consequently, it can be hypothesized that the Carnitine/Acetyl-Carnitine shuttle is completely inactive when YlCrc1 transporter is absent and not compensated by the homologous carrier YALI0A20988g, indicating that in *Y. lipolytica* YlCrc1 is a key component of the sole pathway to transport acetyl-CoA from the cytosol or peroxisomes into mitochondria. To further confirm the subcellular localization of YlCrc1, we constructed an in-frame *CRC1* fusion with RedStar2 as described previously [28]. This plasmid was used to transform the Yl*crc1* knock-out strain. The resulting recombinant strain was able to grow on acetate and oleic acid indicating the functionality of the Yl*CRC1*-RedStar2 fusion. YlCrc1-RedStar2 expressing cells showed a red fluorescence of the mitochondrial network. With the mitochondrial-specific dye, MitoTracker Green, the same cells showed a very similar pattern of fluorescence. From the overlapping image it is clear that YlCrc1-RedStar2 was localized to mitochondria. The structural integrity of the cells was documented by phase contrast microscopy (Additional Fig. 3).

**Fig. 3:**
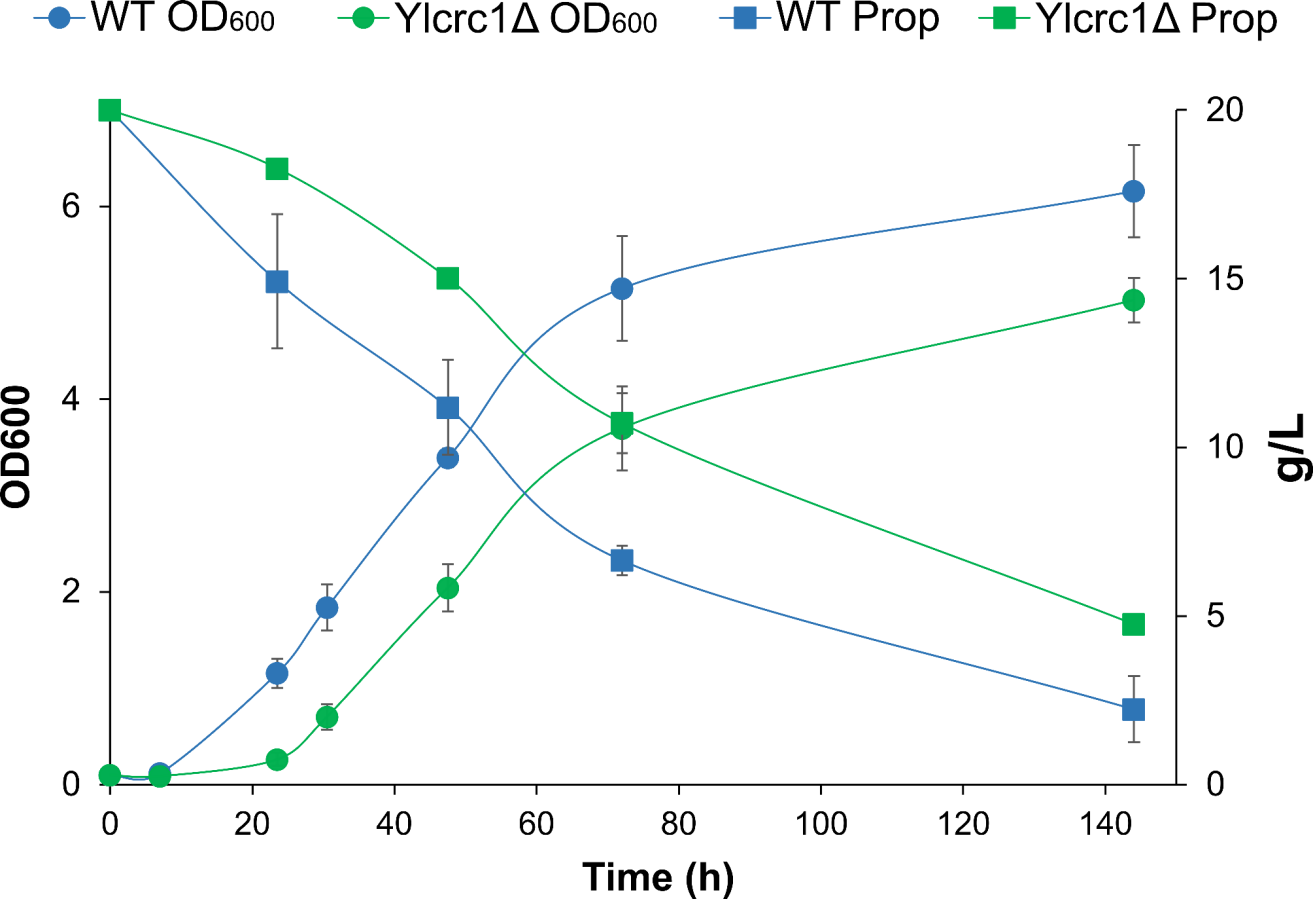
Comparison of growth of the wild type (blue circles) and Yl*crc1*Δ strains (green circles) on YNB medium with propionate (20 g/L) as carbon source and consumption of propionate in wild type (blue squares) and strain with deletion of Yl*CRC1* (green squares). Averages and standard errors were obtained from at least two replicate experiments

Next we investigated whether the deletion of Yl*CRC1* could affect the growth of *Y. lipolytica* on propionate (20 g/L). The growth of Yl*crc1*Δ strain did not differ significantly from that of the wild type (Fig. 3) which produced 3.35 ± 0.37 g DCW/L of biomass while, the deleted strain grew up to 2.69 ± 0.17 g DCW/L. The Yl*crc1*Δ strain only displayed reproducibly a longer lag phase compared to the parental strain. The consumption of propionate was evaluated and in this case, in Yl*crc1*Δ strain it was only slightly lower than that of the wild type (Fig. 3). We concluded that YlCrc1 does not represent the main mitochondrial carrier involved in propionate metabolism in *Y. lipolytica*.

### Identification of putative carnitine acetyltransferases in Yarrowia lipolytica

Carnitine acyl/acetyltransferases in *Y. lipolytica* have not been investigated so far. Protein BLAST analysis using the three CATs of *S. cerevisiae* as queries, revealed two sequences of *Y. lipolytica* encoded by YALI0B10340g and YALI0F21197g which represent potential carnitine acetyltransferases. The amino acidic identity matrix shows that YALI0B10340p and YALI0F21197p have the highest identity with ScCat2p (38.7%) and ScYat1p (48.03%), respectively (Additional Table 2). The multiple alignment of the five amino acid sequences (Additional Fig. 4) revealed for both putative *Y. lipolytica* CATs, the presence of the protein motif at the N-terminal region, LPXLPXPXL, typical of choline and carnitine acetyltransferases [29, 30]. Sc*CAT2* has two in-frame ATG codons, the second of which is located immediately after a mitochondrial targeting signal (MTS) (Additional Fig. 4), predicted by the Mitoprot software [31]. The ScCat2 protein is exclusively directed to peroxisomes when the protein lacks the N-terminal MTS [32]. The peroxisomal localization results from a translational start at the second ATG, and the presence of a functional variant of the peroxisomal targeting signal type 1 (PTS-1) AKL at C-terminal [33, 34] (Additional Fig. 4). Similarly to the *S. cerevisiae* counterpart, YALI0B10340p contains a MTS sequence at N-terminal, a second in-frame ATG codon, after the MTS, and the PTS-1 AKL at the C-terminal (Additional Fig. 4). Hence, YALI0B10340g most likely encodes for a putative carnitine acetyltransferase localized both in peroxisomes and mitochondria, as ScCat2p, and thereof it will be hereafter named YlCat2. The second identified putative *Y. lipolytica* CAT, YALI0F21197p, does not show any mitochondrial or peroxisomal targeting sequence (Additional Fig. 4) and is likely a cytosolic carnitine acetyltransferase like *S. cerevisiae* Yat1p and Yat2p [35]. Due to its highest amino acids identity with ScYat1p (Additional Table 2), we named this protein YlYat1. This analysis shows that unlike *S. cerevisiae*, which has three carnitine acetyltransferases, *Y. lipolytica* most likely has only two CATs which could be homologues of ScCat2p and ScYat1p.

### Expression of Yl*CRC1*, YALI0A20988g and Yl*CAT2* is induced by oleic acid

**Fig. 4:**
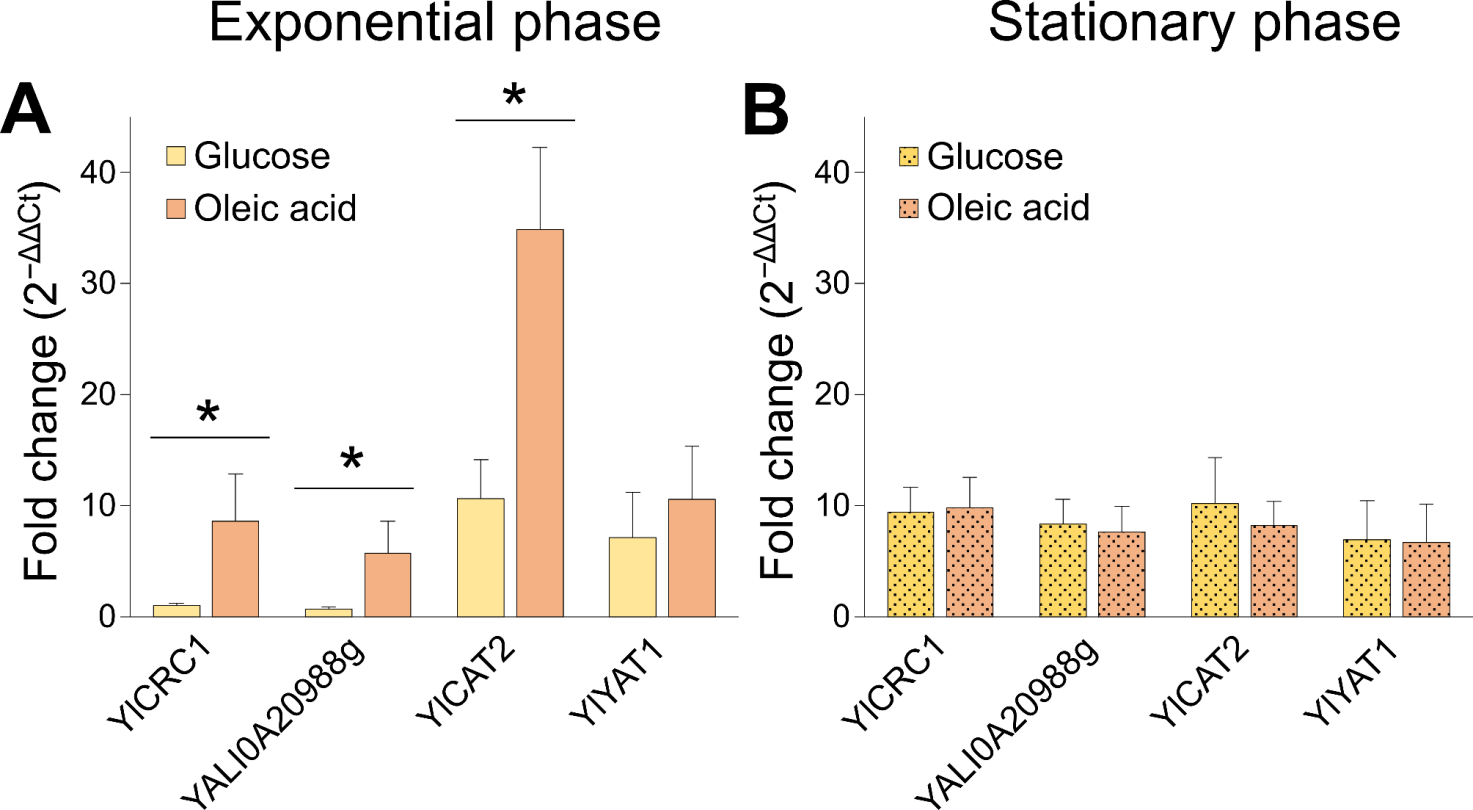
Expression of Yl*CRC1*, YALI0A20988g, Yl*CAT2* and Yl*YAT1* in wild-type cells grown on YNB medium supplemented with glucose (yellow) or oleic acid (orange) (40 g/L) after 15 h (exponential phase) (**A**) and 72 h (stationary phase) (**B**). mRNA levels were quantified by qPCR, and the mRNA of Yl*CRC1* gene, grown on glucose supplemented medium at 15 h, was used as calibrator. The quantification of relative gene expression was calculated according to the comparative method (2^−ΔΔCt^). Averages and standard errors were obtained from at least three independent experiments. * indicates a statistically significant difference in mRNA expression between glucose and oleic acid media (P < 0.05). P value was calculated by unpaired Student’s t-test

The Sc*CRC1* promoter presents an oleate-response element (ORE), found in oleate-inducible yeast genes including those coding for β-oxidation enzymes [36]. This characteristic sequence 5′-CGGN_3_TNAN_9–12_CCG-3′ [37] can be found in the promoter of Yl*CRC1* and YALIA20988g (Additional Fig. 5) that presumably are also regulated by oleic acid. To evaluate the expression levels of the mitochondrial carriers YlCrc1 and YALI0A20988p, and of the two putative CATs YlCat2 and YlYat1, a qPCR analysis was performed in the wild type strain grown on YNB medium supplemented with either 40 g/L glucose or oleic acid as carbon source. The expression level was evaluated in two growth phases, exponential phase (15 h) and stationary phase (72 h) (Fig. 4A-B). During the exponential phase of growth on glucose, the expression of the two putative mitochondrial carnitine carrier homologs is repressed by glucose while, the expression of the two putative CATs, Yl*CAT2* and Yl*YAT1*, is about 7–10 times higher than that of Yl*CRC1* (P < 0.05) (Fig. 4A). The expression of Yl*CAT2* significantly increased by about 3-fold in the oleic acid-containing medium, compared to glucose (P < 0.05), confirming its role in the β-oxidation of FAs. Conversely, Yl*YAT1* expression did not change significantly on oleic acid suggesting a secondary role in fatty acids β-oxidation (Fig. 4A). Also Yl*CRC1* and YALI0A20988g were significantly induced by oleic acid by 9- and 6-fold respectively, compared to glucose-containing medium (P < 0.05) (Fig. 4A), suggesting that acetyl-carnitine transport towards mitochondria can represent a rate-limiting step in β-oxidation.

During the stationary growth phase, the expression levels of both MCs and putative CATs remained constant (Fig. 2B), maybe due to the depletion of the carbon sources. These results strongly support the role of Yl*CRC1* and Yl*CAT2* in the transport of acetyl-CoA from peroxisomes to mitochondria as components of the Carnitine/Acetyl-Carnitine shuttle.

### Role of YlCrc1 in lipid accumulation in Yarrowia lipolytica

**Fig. 5:**
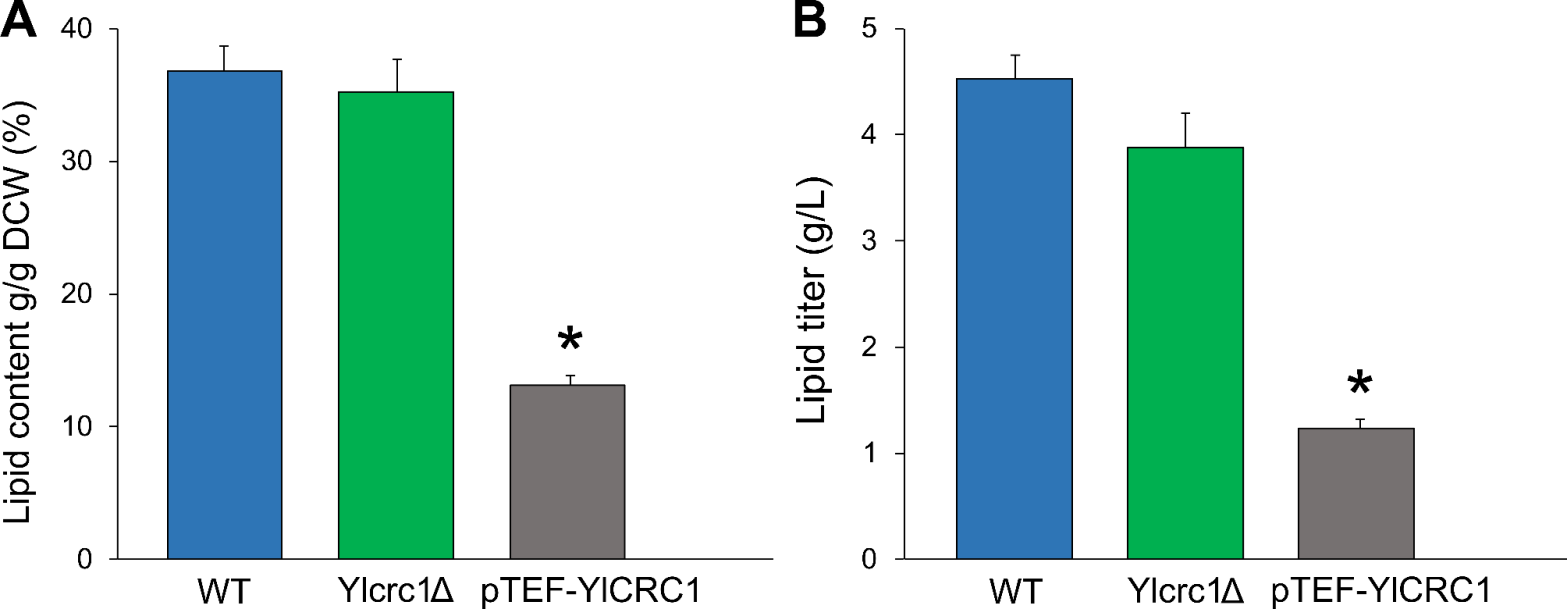
** A**) Cellular lipid content expressed as percentage of fatty acids (FAs) in the total dry cell weight (DCW) and **B**) lipid titer (g/L) of wild type (blue), strain with deletion of Yl*CRC1* (green) and strain overexpressing Yl*CRC1* (grey). The strains were cultivated on YNB + 1.5 g/L NH_4_Cl + 40 g/L glucose medium for 120 h. Averages and standard errors were obtained from two replicate experiments for WT and Yl*crc1*Δ strains and from four replicate experiments for pTEF-Yl*CRC1* strain. * indicates lipid content and lipid titer of pTEF-Yl*CRC1* strain, significantly different from that of WT and Yl*crc1*Δ strains (P < 0.0001 by ANOVA with Fisher’s post hoc test)

To further investigate the role of mitochondrial carrier YlCrc1 in lipid metabolism in *Y. lipolytica*, we overexpressed Yl*CRC1* under the control of TEF promoter in wild type strain (pTEF-Yl*CRC1* strain). The wild type, Yl*CRC1* deletion and overexpressing strains were cultivated in nitrogen-limited conditions (1.5 g/L NH_4_Cl, lipogenic medium) with glucose (40 g/L) for 120 h and lipid accumulation was quantified. In this medium the C/N ratio was 40, compared to 12 found in basic minimal media containing 5 g/L NH_4_Cl and as a consequence, the accumulation of lipids was favored. The wild type and Yl*crc1*Δ strains showed almost the same lipid content of 36.8 ± 1.9% and 35.2 ± 2.5% (g/100 g DCW – dry cell weight) respectively, and lipid productions of 4.53 ± 0.22 and 3.88 ± 0.33 g/L, respectively (Fig. 5). Instead, the overexpression of Yl*CRC1* significantly decreased both lipid content (13.11 ± 0.72%) and lipid titer (1.24 ± 0.08 g/L), compared to the wild type and Yl*crc1*Δ strains (P ≤ 0.0001) (Fig. 5). It was likely that when *Y. lipolytica* was grown on glucose, in a lipogenic medium, a higher amount of acetyl-CoA generated in the cytosol re-entered the mitochondria, due to the overexpression of Carnitine/Acetyl-Carnitine transporter YlCrc1, determining a decrease of the substrate available for the synthesis of lipids.

### Effect of Yl*CRC1* deletion on OCFAs synthesis in *Y. lipolytica*

**Fig. 6:**
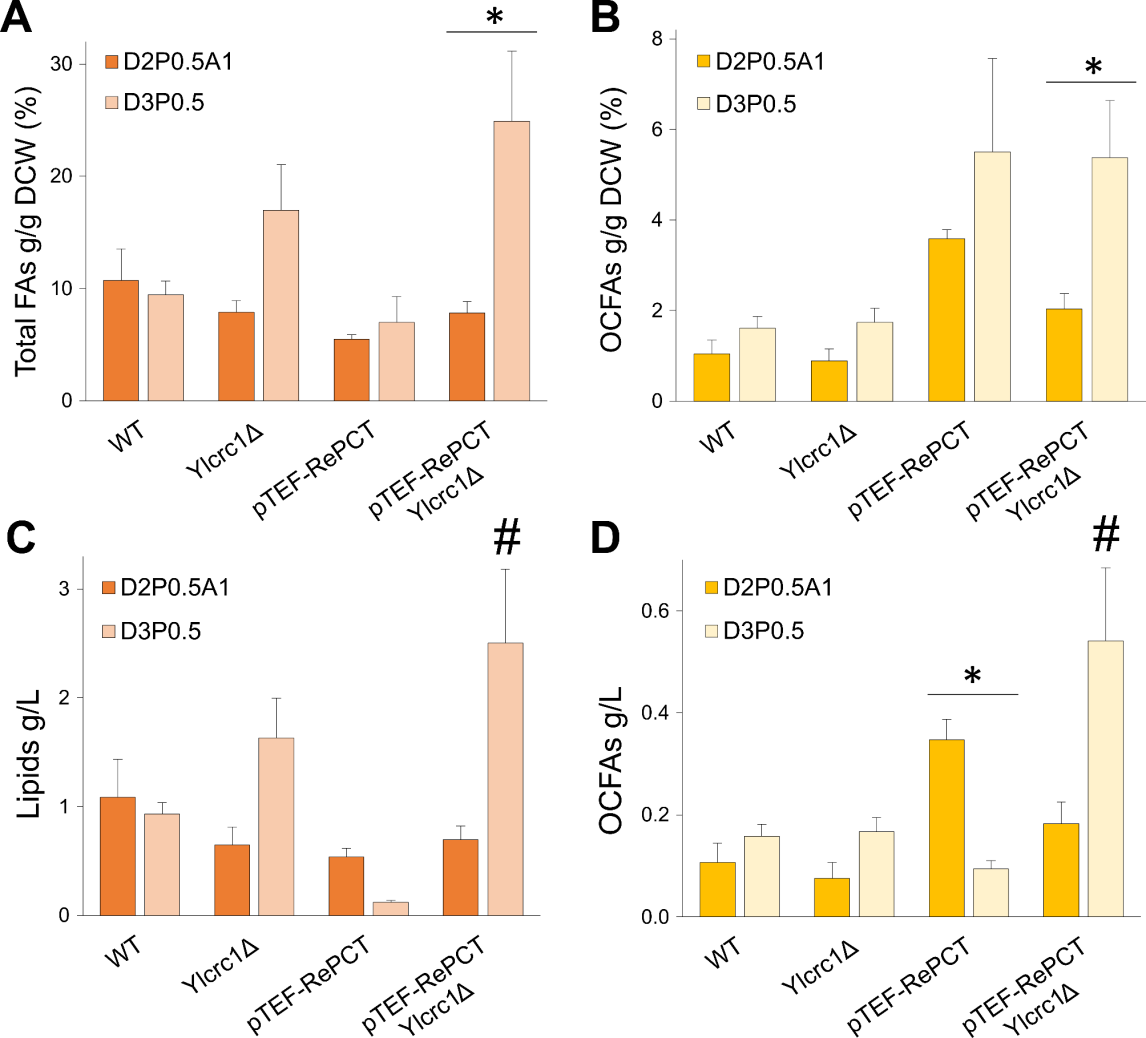
Lipid accumulation in the strains wild type (WT), Yl*crc1*Δ, pTEF-RePCT and pTEF-RePCT Yl*crc1*Δ. **A**) Percentage of total fatty acids and **B**) percentage of OCFAs in the DCW on D2P0.5A1 (orange and yellow) and D3P0.5 (light orange and light yellow) media. **C**) Titer (g/L) of total fatty acids and **D**) total amount (g/L) of OCFAs on D2P0.5A1 (orange and yellow) and D3P0.5 media (light orange and light yellow). Strains were cultivated on nitrogen-limited media (1.5 g/L NH_4_Cl) for 120 h at 28 °C. Averages and standard errors were obtained from at least three replicate experiments. * indicates a statistically significant difference in lipid accumulation between the same strain cultivated on D2P0.5A1 and D3P0.5 media (P < 0.05). **#** indicates a statistically significant difference in lipid and OCFAs titer (g/L) of the pTEF-RePCT Yl*crc1*Δ strain on D3P0.5, compared to other strains (P < 0.0075), except for Yl*crc1*Δ on D3P0.5 (C) and pTEF-RePCT on D2P0.5A1 (D). The significance of the differences was estimated by ANOVA, with Fisher’s post hoc test

The production of OCFAs in *Y. lipolytica* has been obtained using propionate as primer for their synthesis [13]. This three-carbon carboxylic acid was activated to propionyl-CoA, the initial substrate for OCFAs synthesis. The OCFAs production has been further improved, increasing the activation of propionate to propionyl-CoA by overexpressing in *Y. lipolytica* the gene encoding for propionyl-CoA transferase of *Ralstonia eutropha* (RePCT) that catalyzes the transfer reaction of CoA from acetyl-CoA to propionate. This genetic modification increased the accumulation of OCFAs by 3.8-fold over the control strain, with a ratio of OCFAs to total FAs of 53.2% [19]. To evaluate the impact of Yl*CRC1* deletion on OCFAs synthesis two *Y. lipolytica* strains, one overexpressing the RePCT enzyme under the control of TEF promoter (pTEF-RePCT), and another displaying both the overexpression of RePCT and the deletion of the YlCrc1 transporter (pTEF-RePCT Yl*crc1*Δ strain) were constructed. The accumulation of lipids was evaluated after 120 h of shake flask cultures, in nitrogen-limited (1.5 g/L NH_4_Cl) medium D2P0.5A1 (glucose 20 g/L; propionate 5 g/L and acetate 10 g/L) [19]; the lipid profile (% of fatty acids) is reported in Additional Table 3. The addition of acetate to the medium, has been previously found to be necessary to restore a proper growth of the RePCT-overexpressing strain, in the presence of glucose and propionate as carbon sources [19]. On D2P0.5A1 medium, the strain pTEF-RePCT Yl*crc1*Δ displayed a slightly increased production of OCFAs compared to the wild type (0.18 ± 0.04 vs. 0.11 ± 0.04 g/L) (Fig. 6D). However, this strain showed a 2-fold lower percentage of OCFAs compared to pTEF-RePCT strain (Fig. 6B-D). In this strain the most abundant fatty acid was cis-9-heptadecenoic acid (C17:1–53.8 ± 1.71%) (Additional Table 3). These data led us to hypothesize that the deletion of Yl*CRC1* results in a higher concentration of cytosolic acetyl-CoA which, competes with propionyl-CoA determining a decrease of OCFAs production. Consequently, we changed the composition of nitrogen-limited medium removing acetate: the obtained medium was called D3P0.5 (lipogenic medium containing glucose 30 g/L and propionate 5 g/L). Glucose concentration was increased in order to maintain the same total amount of carbon sources compared to D2P0.5A1. The removal of acetate increased the lipid accumulation of the strains carrying the Yl*CRC1* deletion: lipid content in the biomass increased by *2*-fold in the Yl*crc1*Δ strain and significantly increased by 3-fold in the pTEF-RePCT Yl*crc1*Δ strain, compared to the medium in which acetate was present (P = 0.0003) (Fig. 6A). In contrast, in the D3P0.5 medium the pTEF-RePCT strain produced only 0.12 ± 0.02 g/L of fatty acids (Fig. 6C) as a consequence of its defect in these growth conditions, in agreement with what was previously reported [19], probably determined by a low acetyl-CoA availability. In fact, only 2.01 ± 0.30 g DCW/L of biomass was obtained for the RePCT-expressing strain (Table 1), after 120 h of growth on D3P0.5 medium; on the contrary, the supplementation of acetate allowed the biomass of the pTEF-RePCT strain to reach 9.71 ± 1.01 g DCW/L (Table 1). The deletion of Yl*CRC1* in the RePCT overexpressing strain (pTEF-RePCT Yl*crc1*Δ) determined a significant restoration of the growth (9.98 ± 0.40 g DCW/L) also in the absence of acetate (D3P0.5 medium) (Table 1). The percentage of OCFAs content increased about 2-fold for the Yl*crc1*Δ strain and significantly increased by 3-fold for the pTEF-RePCT Yl*crc1*Δ strains, in the medium without acetate, compared to the medium with acetate (P = 0.003) (Fig. 6B), probably as a consequence of a higher propionyl-CoA/acetyl-CoA ratio in the cytosol. In the absence of acetate in the growth medium (D3P0.5) the pTEF-RePCT strain accumulated only 0.09 ± 0.02 g/L of OCFAs, a titer 4-fold lower than that obtained on medium with acetate (P < 0.05) (Fig. 6D) despite the very high OCFA cellular content with C17:1 reaching the 64.33 ± 6.03% of all the fatty acids (Additional Table 3). Among the tested strains, the highest and significant OCFAs titer was achieved with pTEF-RePCT Yl*crc1*Δ strain, which produced 0.54 ± 0.14 g/L of OCFAs on D3P0.5 (P < 0.01) (Fig. 6D). This OCFAs titer is 1.5-times higher than that of pTEF-RePCT strain on D2P0.5A1 medium.

These results indicate pTEF-RePCT Yl*crc1*Δ as a promising platform for the construction of industrial OCFAs producing strains, allowing higher production titers and a simplification of the growth medium.

**Table 1:** Biomass (g DCW/L) of strains cultivated in two nitrogen-limited (1.5 g/L NH4Cl) media: D2P0.5A1 (glucose 20 g/L; propionate 5 g/L and acetate 10 g/L) and D3P0.5 (glucose 30 g/L and propionate 5 g/L) for 120 h. Values are reported as average ± standard error from at least three replicate experiments. ** indicate a statistically significant difference (P < 0.01) estimated by a Student’s t-test

g DCW/L		
	D2P0.5A1	D3P0.5
WT	9.73 ± 0.62	9.92 ± 0.38
Yl*crc1*Δ	7.97 ± 1.13	9.64 ± 0.15
pTEF-RePCT	9.71 ± 1.01	2.01 ± 0.30 **
pTEF-RePCT Yl*crc1*Δ	8.62 ± 0.50	9.98 ± 0.40

## Discussion

Based on sequence comparison, we have identified the *Y. lipolytica* gene YALI0C02431, as the closest homolog of *S. cerevisiae* gene which encodes for the MC ScCrc1, and named it YlCrc1. The amino acid sequence of YlCrc1 showed the presence of the three characteristic signature motifs of MCs (PX[DE]XX[RK]X[RK]) on the hydrophobic transmembrane segments H1, H3 and H5 [9]. Furthermore, the presence of the distinct motif RXXPANAAXF of CAC carriers at the end of the sixth hydrophobic domain, involved in conformational changes and substrate binding during Carnitine/Acetyl-Carnitine translocation [26], made consistent the hypothesis that YlCrc1 might be involved in Carnitine/Acetyl-Carnitine shuttle. Real-time qPCR experiments confirmed that Yl*CRC1* is more expressed when oleic acid is used as carbon source, compared with glucose (Fig. 4A), as suggested by the presence of an oleate-response element in the promoter of Yl*CRC1*, indicating that YlCrc1 plays a role in β-oxidation.

In order to investigate the function of YlCrc1 in *Y. lipolytica* metabolism, we characterized the strain carrying deletion of Yl*CRC1* gene. We confirmed the ability of Yl*crc1*Δ strain to grow on glucose-based media (Fig. 2A), excluding the role of YlCrc1 in metabolism of carbohydrates-like substrates as already found for the *S. cerevisiae* counterpart [38]. Instead, differently from what found for *S. cerevisiae*, the deletion of Yl*CRC1* impaired the ability to grow on acetate, ethanol and oleic acid (Fig. 2B-C-D), indicating a key role of this transporter in shuttling acetyl-CoA generated in cytosol or peroxisome to the mitochondrial matrix. In *S. cerevisiae* two pathways by which, cytosolic or peroxisomal acetyl-CoA can reach the mitochondrial matrix for its complete oxidation to CO_2_ have been reported [39]. Acetyl-CoA can fuel the peroxisomal glyoxylate cycle producing succinate which is then transported to mitochondria. Alternatively, acetyl-CoA is reversibly converted into acetyl-L-carnitine and translocated across the mitochondrial membrane by ScCrc1 [11, 37]. Consequently, in *S. cerevisiae* only the double deletion of Sc*CRC1* and Sc*CIT2*, encoding the peroxisomal citrate synthase, strongly impairs the growth of cells on oleic acid leading to the arrest of the β-oxidation [38]. Conversely, the inability of Yl*crc1*Δ strain to grow on oleic acid, acetate and ethanol, strongly indicates that *Y. lipolytica*, at least in the tested growth conditions, can use only the Carnitine/Acetyl-Carnitine shuttle to transport cytosolic and peroxisomally generated acetyl-CoA to mitochondria. This result is quite surprising considering the much higher ability of *Y. lipolytica* to oxidize acetyl-CoA yielding substrates than *S. cerevisiae*.

In *S. cerevisiae* the activation of propionate to propionyl-CoA can be catalyzed by Acs1, a cytosolic isoenzyme of acetyl-CoA synthetase [40]. It has been reported that in *S. cerevisiae*, propionyl-CoA is catabolyzed in the mitochondrial matrix through the methylcitrate pathway [41], which is also active in *Y. lipolytica* [42]. In *vitro* experiments carried out on the recombinant ScCrc1 reconstituted in liposomes, have showed that this mitochondrial carrier is able to transport, propionyl-L-carnitine in exchange with L-carnitine [11]. The high percentage of amino acids identity among ScCrc1 and YlCrc1 (50%) led us to speculate a role of YlCrc1 in propionate catabolism. The disruption of Yl*CRC1* gene did not affect the growth of *Y. lipolytica* on propionate, as well as its consumption (Fig. 3), demonstrating that YlCrc1 does not have a major role in propionate metabolism and suggesting the presence of another mitocondrial carrier responsible for the uptake of propionyl-L-carnitine into the mitochondria. Interestingly, we identified the gene YALIA20988 as a close homolog of Yl*CRC1* (Fig. 1). In this MC, the characteristic motif of the hitherto identified CAC transporters is only partially conserved and the amino acids identity with the known CAC carriers is much lower than that of YlCrc1. Yet, an ORE can also be found in the promoter of YALIA20988g which results in the induction of its expression by oleic acid, as measured by real-time qPCR (Fig. 4A). Experiments are ongoing to test whether the disruption of YALI0A20988g has an impact on propionate utilization by *Y. lipolytica*.

Besides the mitochondrial carrier YlCrc1, we identified the two putative carnitine acetyltransferase enzymes YlCat2 and YlYat1, encoded by YALI0B10340g and YALI0F21197g, respectively; no other *Y. lipolytica* protein displayed a significant homology with the three known *S. cerevisiae* CAT isoenzymes. YlCat2 has the higher amino acid identity with ScCat2 and presents a MTS at the N-terminus, a second in-frame ATG codon placed after the MTS, and a PTS-1 at the C-terminus (Additional Fig. 4). These elements are fundamental for the dual localization of ScCat2 in peroxisomes and mitochondria [32]. Moreover, the expression of Yl*CAT2* is strongly induced by oleic acid (Fig. 4A), in analogy with the higher ScCat2 activity detected in cells grown on oleic acid [32]. Even if Yl*CAT2* is induced by oleate, its induction level (3.3 fold) is much lower than that of Yl*CRC1*, suggesting a possible role of the carrier on the control of the rate of the carnitine shuttle. YlYat1 is more similar to ScYat1 and ScYat2 and, the lack of mitochondria and/or peroxisomes targeting signals, suggests a cytoplasmic localization as demonstrated for ScYat2 [35]. No induction of Yl*YAT1* significantly exceeding the level observed in glucose medium was seen, with oleic acid as the sole carbon source (Fig. 4A). The same observation was done for Sc*YAT1* [43]. This finding makes it very unlikely that Yl*YAT1* codes for a peroxisomal oleic acid-inducible enzyme, strongly supporting its role in ethanol and acetate metabolism that occurs in the cytoplasm.

**Fig. 7:**
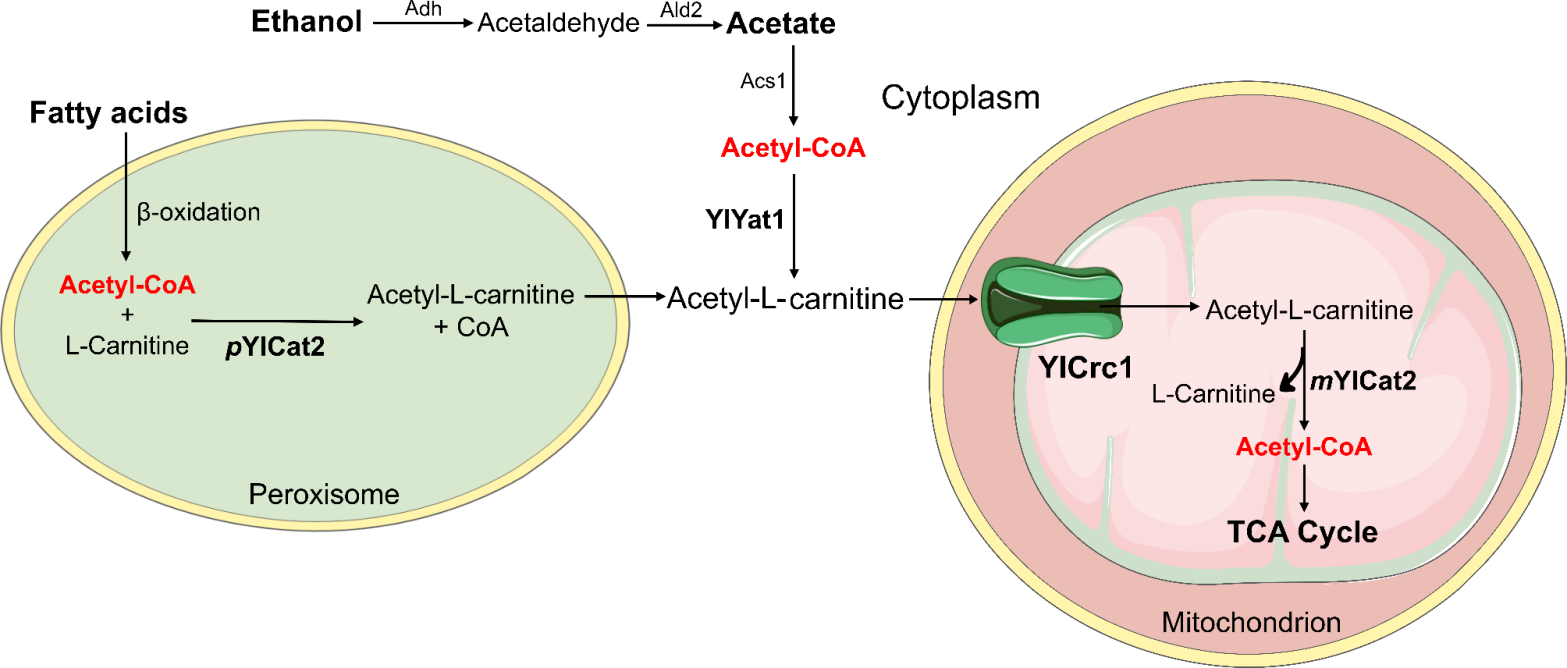
Proposed scheme for metabolism and transport of acetyl groups among peroxisomes, cytosol and mitochondria during growth of *Y. lipolytica* on non-fermentable substrates. Alcohol dehydrogenase (Adh), Aldehyde dehydrogenases (Ald2), Acetyl-CoA synthetase (Acs1), peroxisomal form of CAT (pYlCat2), cytoplasmic CAT (YlYat1) and mitochondrial form of CAT (mYlCat2). In green the mitochondrial carrier YlCrc1. The figure was produced using the vector image bank of Servier Medical Art (http://smart.servier.com/)

Based on the results reported in this work, we propose a model for metabolism and transport of acetyl groups among peroxisomes, cytosol and mitochondria during growth of *Y. lipolytica* on non-fermentable substrates (Fig. 7). During growth on ethanol, acetate, or fatty acids, acetyl units must enter the mitochondria for metabolism via the TCA cycle. Acetyl-CoA is produced in the cytoplasm by acetyl-CoA synthetase Acs1 [7] during growth on acetate and ethanol while for β-oxidation of fatty acids, acetyl-CoA is synthesized in peroxisomes. The peroxisomal form of carnitine acetyltransferase pYlCat2 and the cytoplasmic CAT YlYat1, reversibly convert acetyl-CoA into acetyl-L-carnitine. Then, acetyl-L-carnitine enters the mitochondrial matrix, transported by the mitochondrial carrier YlCrc1. The mitochondrial form of carnitine acetyltransferase mYlCat2 catalyses the reverse reaction to form carnitine and acetyl-CoA which enters the TCA cycle for its complete oxidation.

In order to investigate the role of the mitochondrial carrier YlCrc1 in lipid metabolism in *Y. lipolytica*, we verified whether the deletion of Yl*CRC1* would impact the production of FAs. On glucose-based medium, the deletion of the MC did not increase lipids; while Yl*CRC1* overexpression significantly reduced the lipid content by approximately 3-fold, compared to the wild type (Fig. 5). This result further suggest that YlCrc1 can represent a key point for the regulation of FAs metabolism and for the balancing of FA synthesis and their oxidation.

The overexpression of Yl*CRC1* in *Yarrowia lipolytica* probably determines when cells are grown on glucose, a futile cycle: the increased import of cytosolic acetyl-CoA in mitochondria, could enhance mitochondrial citrate synthesis and accumulation (due to citric acid cycle slowdown under nitrogen starvation). Citrate could then be transported to the cytosol to be converted into acetyl-CoA by ATP citrate lyase (ACL) restoring cytosolic acetyl-CoA levels. Experiments are ongoing to determine if the expression levels of this carrier can modulate the pool of acetyl-CoA in the cytoplasm, a crucial precursor of several biotechnologically important products. All data presented in this work strongly support the role of YlCrc1 as the main Carnitine/Acetyl-Carnitine carrier in *Y. lipolytica*.

Besides the functional characterization of *Y. lipolytica* mitochondrial carrier YlCrc1, we sought to apply the acquired knowledge in a metabolic engineering approach aiming at improving odd-chain fatty acids (OCFAs) synthesis. In *Y. lipolytica*, OCFAs production is enhanced by the use of propionate as primer since, after its activation to propionyl-CoA, it replaces acetyl-CoA in the first condensation step of FAs synthesis [13]. It has been previously reported that a strategy to improve OCFAs production in *Y. lipolytica*, is the heterologous expression of the enzyme RePCT (propionyl-CoA transferase of *Ralstonia eutropha*), enhancing the activation of propionate to propionyl-CoA [19]. The authors observed that to support the synthesis of OCFAs by the RePCT-overexpressing strain, besides propionate, it was essential to add acetate to the lipogenic medium (synthetic medium with a high C/N ratio) in order to avoid a slower growth. The high activity of RePCT which catalyzes the transfer of CoA group from acetyl-CoA to propionate, depletes cytosolic acetyl-CoA levels impairing the cellular growth. For this reason, they optimized the medium for OCFAs synthesis by using glucose, propionate and acetate (D2P0.5A1 medium) [19]. We evaluated the impact of both the deletion of Yl*CRC1* and the overexpression of RePCT on OCFAs production, by using the lipogenic medium D2P0.5A1. The OCFAs titer still remained 2-fold higher in the RePCT-expressing strain than in the pTEF-RePCT Yl*crc1*Δ strain (Fig. 6D). The decrease in OCFAs cellular content in the pTEF-RePCT Yl*crc1*Δ strain was attributed to a possible higher acetyl-CoA cytosolic accumulation caused by the deletion of Yl*CRC1*, determining a lower propionyl-CoA/acetyl-CoA ratio. For this reason, we decided to remove acetate from the lipogenic medium and to utilize only glucose and propionate 30 g/L and 5 g/L, respectively (D3P0.5 medium). In the absence of acetate, pTEF-RePCT Yl*crc1*Δ strain outperformed the RePCT-expressing strain since, we were able to restore the growth on a medium containing only glucose and propionate (Table 1), still maintaining high OCFAs titer (Fig. 6D). In this medium, the cytosolic acetyl-CoA was not limiting for the growth, probably due to a rebalancing of the propionyl-CoA/acetyl-CoA ratio. Furthermore, the removal of acetate and the concomitant deletion of Yl*CRC1* increased the accumulation of total FAs in both Yl*crc1*Δ and pTEF-RePCT Yl*crc1*Δ strains (Fig. 6A-C). The presented data suggest that in the D3P0.5 medium there is an increase of lipid re-consumption compared to D2P0.5A1 and that the deletion of Yl*CRC1* prevents it. Yet, the qPCR revealed no significant differences in the expression levels of Yl*CRC1* or of Yl*CAT2*, on G2P.05A1 vs. D3P0.5 media (Additional Fig. 6); hence, the molecular mechanisms explaining this observation deserves further investigation. As a consequence of the increased lipid accumulating capacity on the D3P0.5 medium, the pTEF-RePCT Yl*crc1*Δ strain represents a very promising platform for the synthesis of OCFAs.

## Conclusions

In this study, we have identified the mitochondrial carrier involved in Carnitine/Acetyl-Carnitine shuttle in *Yarrowia lipolytica* (YlCrc1) and the two most likely carnitine acetyltransferases YlCat2 and YlYat1. Our findings suggest that this shuttle is the main pathway used by *Y. lipolytica*, for fatty acids utilization and transport of acetyl-CoA from peroxisome and the cytosol to mitochondria. A metabolic engineering approach, based on the deletion of the mitochondrial carrier Yl*CRC1*, along with the overexpression of the enzyme RePCT, allowed the simplification of the lipogenic medium for OCFAs synthesis and improved their accumulation in *Y. lipolytica*. This work further proves how metabolic fluxes can be deeply affected by the manipulation of the expression levels of mitochondrial carriers.

## Materials and methods

### Media and culture conditions

*Escherichia coli* DH5α strains were grown at 37 °C in LB medium (1% tryptone, 0.5% yeast extract, 1% NaCl w/v) supplied with 50 µg/mL kanamycin or 100 µg/mL ampicillin (Sigma-Aldrich, Missouri, USA) for cloning and plasmid propagation. *Y. lipolytica* strains were cultivated at 28 °C in YPD medium (1% yeast extract, 2% peptone, 2% glucose w/v) for growth and synthetic YNB medium (0.17% yeast nitrogen base without amino acids, 0.5% NH_4_Cl, 2% glucose w/v and 50 mM phosphate buffer pH 6.8) for screening of transformants. When necessary, YNB medium was supplemented with 100 mg/L uracil and/or leucine for strain auxotrophy complementation. The YNB media containing different carbon sources were named according to the substrate (D = glucose; P = sodium propionate; A = sodium acetate; E = ethanol) followed by the concentration (in % w/v or v/v for ethanol) of the compounds. In order to obtain a C/N ratio of 30–40 in YNBD2P0.5A1 and YNBD3P0.5, 0.15% NH_4_Cl (w/v) was used. Solid media were prepared by adding 1.5% agar (w/v) to liquid media. In YNBOA solid media, 1% oleic acid emulsion (from a 20% oleic acid, 0.5% Tween 40 v/v stock solution) was added in YNB medium. All cultures were maintained at – 80 °C in glycerol stock.

### Strains and plasmids construction

All the plasmids and *Y. lipolytica* strains used in this study are reported in Table 2. Primers used in this study are listed in Additional Table 4. Standard molecular genetic techniques were carried out as previously described [44]. Restriction enzymes, ligases and kinases were obtained from New England Biolabs (NEB) (Massachusetts, USA) or Thermo Scientific (Vilnius, Lithuania). PCR amplifications were performed using Q5 High-Fidelity DNA Polymerase Master Mix (NEB, Massachusetts, USA) or GoTaq Green Master Mix (Promega, Wisconsin, USA). Oligonucleotides were provided by Eurofins Genomics (Ebersberg, Germany). Plasmids were extracted using the NucleoSpin Plasmid EasyPure kit (Macherey-Nagel, Düren, Germany). All reactions were performed according to the manufacturer’s instructions. The Yl*CRC1* gene deletion was carried out using a combinatorial dual cleavage CRISPR/Cas9-mediated [27], through paired gRNAs targeting upstream and downstream sites of Yl*CRC1* gene. To this end, 20 bp target sequences located to 150 and 250 bp upstream and downstream of the start codon, respectively, were predicted based on *Y. lipolytica* W29 genome and cloned into JME4390 and JME4472 replicative plasmids [45]. Assembled CRISPR/Cas9 vectors were used to co-transform *Y. lipolytica* strains by the lithium acetate method [46] and YNB solid medium was used to select transformants. The verification of Yl*CRC1* disruption was performed by colony PCR, amplifying the genomic region targeted by the two gRNAs and comparing the amplicons length of the transformants, with that obtained from the parental strains. In addition, these amplicons were purified (NucleoSpin PCR Clean-up kit, Macherey-Nagel, Düren, Germany) and sequenced (Mix2Seq, Eurofins Genomics, Ebersberg, Germany) to confirm the disruption of the CDS of Yl*CRC1* gene. For Yl*CRC1* homologous gene overexpression, the Yl*CRC1* coding sequence flanked by AvrII(XmaJI) and BamHI restriction sites was amplified from *Y. lipolytica* W29 genome and cloned into JME5574 plasmid – harboring TEF promoter, LIP2 terminator and URA3ex expression cassette as auxotrophy marker (Vidal unpublished). Expression vector was digested by NotI and used to transform *Y. lipolytica* strains. To overexpress RePCT gene, strains were transformed with NotI linearized JME4070 (pTEF-RePCT + URA3ex) [47] and gene integration was verified by colony PCR. To restore URA3 or LEU2 prototrophy, strains were transformed with NotI linearized JME1046 (URA3ex) or JME2563 (LEU2ex) plasmids, respectively [48, 49]. For the marker rescue procedure, strains were transformed with JME0461 replicative plasmid for the transient expression of CRE recombinase [50]. Replicative plasmids were lost by consecutive cultures in YPD rich media and checked by plating in minimal media with and without auxotrophic supplement. For subcellular localization experiment, the coding sequence of *CRC1* was amplified by PCR from *Y. lipolytica* genomic DNA without termination codons and with additional BamHI and AvrII restriction sites. The product was cloned into the JME1432 vector in frame with the RedStar2 (Red fluorescent protein) coding sequence, as described previously [28]. Expression vector was digested by NotI and used to transform *Y. lipolytica* strain JMY9118 (Table 2).

**Table 2:** Plasmids and strains used in this study

Plasmids	Description	References
JME0461	CRE-*LEU2*	[50]
JME1046	JMP62-*URA3*ex-pTEF	[48]
JME1432	pTEF-*RedStar2*-Cter-URA3	Nicaud & Haddouche (unpublished)
JME2563	JMP62-*LEU2*ex-pTEF	[49]
JME4070	JMP62-*URA3ex*-pTEF-*RePCT*	[47]
JME4390	GGA_*LEU2*ex_CrisprCas9-yl_RFP	[45]
JME4472	GGA_*URA3*ex_CrisprCas9-yl_RFP	[45]
JME5627	JME4472 + gRNAYl*CRC1-1*	This work
JME5628	JME4390 + gRNAYl*CRC1-2*	This work
JME5574	JMP62-*URA3*ex-pTEF-RFP	Vidal (unpublished)
JME5646	JME5574 + *URA3*ex-pTEF-Yl*CRC1*	This work
p-M4B361	JME1432-pTEF-Yl*CRC1*-*RedStar2*-URA3	This work
***Y. lipolytica*** **strains**		
JMY2394	MatA *leu2-270 ura3-302 xpr2-322 ku70*Δ	[51]
YM4B271 (WT)	JMY2394 + *URA3*ex + *LEU2*ex	This work
JMY9067	JMY2394 + *URA3*ex-pTEF-*RePCT*	This work
YM4B272 (pTEF-RePCT)	JMY9067 + *LEU2*ex	This work
JMY9118	JMY2394 Yl*crc1*Δ	This work
YM4B273 (Yl*crc1*Δ)	JMY9118 + *URA3*ex + *LEU2* ex	This work
JMY9095	JMY9067 + pTEF-*RePCT*	This work
JMY9130	JMY9095 Yl*crc1*Δ	This work
YM4B274 (pTEF-RePCT Yl*crc1*Δ)	JMY9130 + *URA3*ex + *LEU2*ex	This work
JMY9097	JMY2394 + *URA3*ex-pTEF-Yl*CRC1*	This work
YM4B2121 (pTEF-Yl*CRC1*)	JMY9097 + *LEU2*ex	This work
YM4B2122 (Yl*crc1*Δ + Yl*CRC1*)	JMY9118 + *URA3*ex-pTEF-Yl*CRC1* + *LEU2*ex	This work
YM4B361	JMY9118 + *URA3*-pTEF-Yl*CRC1*-*RedStar2*	This work

### Cultures conditions for growth analysis

For growth analysis, initial pre-cultures were grown into tubes containing 3 mL of YPD medium and cultured overnight at 28 °C and 180 rpm. Pre-cultures were then centrifuged and washed with sterile distilled water and inoculated in 50 mL shake flasks containing 30 mL of fresh YNBD2, YNBP2, YNBA2 and YNBE2 media, with an initial optical density at 600 nm (OD_600nm_) = 0.1 and cultivated at 28 °C and 180 rpm for 144 h. Growth was monitored by measuring the OD_600nm_ values every 24 h. To verify the ability of transformants to oxidize fatty acids, a drop test was performed using YNBOA plates incubated at 28 °C for 72 h. All cultures were carried out at least in duplicate and average and standard error values were calculated.

### Culture conditions for lipid accumulation

For determining lipid accumulation, an initial pre-culture was established by inoculating a single colony in 10 mL of YPD medium and grown overnight at 20 °C and 180 rpm. The resulting cell suspension was washed with sterile distilled water and used to inoculate 50 mL of YNB medium containing 0.15% (w/v) NH_4_Cl and 50 mM phosphate buffer (pH 6.8) with various concentrations of carbon sources, in 250 mL Erlenmeyer flasks, with an initial OD_600nm_ = 0.1. The cultures were maintained at 28 °C and 140 rpm for 120 h. To determine dry cell weight (DCW), the cultures were washed, frozen and lyophilized in a pre-weighted tube. The differences in weight corresponded to the mg of cells found in the dried culture volume. For each data, at least three biological replicates were used to calculate average and standard error values.

### Microscopy

To test the subcellular localization of YlCrc1, YM4B361 cells were grown overnight in 10 mL of glycerol-supplemented YNB medium (20 g/L). Then, 4 OD of growing cells were harvested and washed with sterile water and with fresh YNB medium, without carbon source. 500 µl of cells were incubated for 30 min at 28 °C and 650 rpm in a ThermoMixer, in the presence of 200 nM MitoTracker Green FM (Molecular Probes, Leiden, The Netherlands), washed with fresh medium and imaged at the microscope. We employed a Zeiss Axiovert 200 inverted epifluorescence microscope equipped with a Plan-Neofluar 100×/1.30 Ph3 oil objective (Carl Zeiss Jena, Germany), and with a CoolSNAP HQ CCD camera (Roper Scientific, Trenton, NJ, USA) using the Metamorph software (Universal Imaging Corporation, Downington, PA, USA).

### RNA extraction and quantitative reverse transcription PCR (qPCR)

The wild type strain was grown on YNBD4, YNBOA4, YNBD2P0.5A1 and YNBD3P0.5 media. Total RNA extraction was performed harvesting 3 OD of exponentially (15 h) and stationary (72 h) growing cells. Cell pellets were resuspended in 1 ml of buffer (Sorbitol 1 M, EDTA 100 mM and 0.1% 2-Mercaptoethanol) containing Zymolyase 0,04 U/ml (amsBIO, UK) and incubated at 30 °C for 1 h. The suspensions were centrifuged, and the pellets were subjected to mechanical disruption with glass beads (SIGMA-ALDRICH, USA) in the bead beating grinder MM200 (Retsch, Verder Scientific GmbH & Co. KG) over 3 cycles at 25 Hz/min for 30 s, and 30 s on ice. Then, RNA was extracted from lysate using the Aurum Total RNA kit (BioRad, Hercules, CA, USA) according to the manufacturer’s instructions. An additional in-column DNase I treatment was included to ensure complete removal of DNA. The amount of RNA was determined by measuring the absorbance at 260 nm with NanoDrop 1000 (Thermo Fisher Scientific, Waltham, MA, USA) and quality was assessed by the 260/280 absorbance ratio with values of 1.8–2.0. Extracted RNA was reverse transcribed using iScript Reverse Transcription Supermix kit (BioRad, Hercules, CA, USA) with mix of random hexamers and oligo (dT) as primers, according to the standard protocol. qPCR was performed in triplicate using the QuantStudio 3 Real-Time PCR System (Applied Biosystems, Thermo Fisher Scientific, Waltham, MA, USA). The primer sequences designed with Primer Express 3.0 (Applied Biosystems, Thermo Fisher Scientific, Waltham, MA, USA) and purchased from Eurofins (Italy), are listed in Additional Table 4. The total reaction volume was 20 µl containing 25 ng of reverse transcribed first-strand cDNA, 10 µl of Sybr Select Master Mix (Applied Biosystems, Life Technologies) and 300 nM of each primer. The specificity of the PCR amplification was checked with the heat dissociation protocol after the final cycle of PCR. To correct for differences in the amount of starting cDNAs, the TPI1 mRNA [52] was amplified in parallel as a reference gene. The relative quantification of the investigated genes was performed according to the comparative method (2^−ΔΔCt^) [53, 54]. The value of 2^− ΔΔCt^ indicates the fold change in gene expression relative to Yl*CRC1* mRNA of cells grown in medium containing glucose at exponential phase as the calibrator.

### Analytical methods

Glucose, propionate, acetate and ethanol were identified and quantified by HPLC, using a Waters Alliance 2695 separation module (Waters, Milford, MA, USA) equipped with a Resex ROA-Organic Acid H^+^ (8%) 300 mm x 7.8 mm column (Phenomenex Inc., Torrance, CA, USA), coupled to a Waters 2410 refractive index detector and a Waters 2996 UV detector. Separation was carried out at 60 °C with 0.0025 M H_2_SO_4_ as mobile phase at a flow rate of 0.5 mL/min. Samples were identified by comparing the retention times with those of standards.

Lipids were extracted from 10 to 30 mg of freeze-dried cells and converted into FA methyl esters (FAMEs) [55]. The FAMEs were then analyzed using gas chromatography (GC), with a Varian 3900 instrument (Varian Inc. USA) [19]. The FAMEs of even-chain fatty acids were identified via comparisons with commercial standards (FAME32, Supelco). Commercial standards of OCFAs (Odd Carbon Straight Chains Kit containing 9 FAs, OC9, Merck, Germany) were converted into their FAMEs using the method previously described [55]. They were then analyzed by GC to identify the OCFAs from the yeast samples. For fatty acids quantification, dodecanoic acid (C12:0) (Sigma-Aldrich, USA), was used as internal standard. Fatty acids were quantified by normalization to internal standard. For each data point, at least three biological replicates were used, and average and standard error values were calculated.

### Bioinformatic analysis

The phylogenetic tree was constructed using 39 protein sequences of *Y. lipolytica* and 37 of *S. cerevisiae*, displaying the mitochondrial carrier family characterizing domain (PFAM number PF00153). The phylogenetic tree was designed using PhyML v3.1 in seaview4 [56] from a Muscle multiple-sequence alignment and drawn in iTOL v.6 [57]. The genes coding for putative carnitine acetyltransferase (CAT) of *Y. lipolytica* were identified by on-line BLASTp search [58] using the sequences of *S. cerevisiae* CATs as queries. Amino acid sequences were aligned by on-line tool NPS@: Network Protein Sequence Analysis [59]. The identity matrix were created by using the web tool SIAS Sequence Identity And Similarity [60]. The prediction of mitochondrial targeting signal (MTS) on amino acid sequences, was carried out by Mitoprot software [31].

## Electronic supplementary material

Below is the link to the electronic supplementary material.

**Additional Tables: Additional Table 1.** Identity Matrix of CAC mitochondrial carriers. Percentage of amino acidic identity among Carnitine/Acetyl-Carnitine mitochondrial carriers of *Saccharomyces cerevisiae* (ScCrc1), *Aspergillus nidulans* (AnCac), *Homo sapiens* (HsCact) and the two putative CAC transporters of *Yarrowia lipolytica* (YlCrc1 and YALI0A20988p). The identity matrix was created by using the web tool “SIAS Sequence Identity And Similarity”. **Additional Table 2.** Identity Matrix of CAT enzymes of *S. cerevisiae* and *Y. lipolytica*. Percentage of amino acids identity among carnitine acetylcarnitine transferases (CATs) of *S. cerevisiae* (Cat2, Yat1 and Yat2) and the two putative CAT enzymes of *Y. lipolytica* (YALI0B10304p and YALI0F21197p). The identity matrix was created by using the web tool “SIAS Sequence Identity And Similarity”. **Additional Table 3.** Lipid profile of *Y. lipolytica* strains. Lipid profile (% of total lipids) of wild type (WT), Yl*crc1*?, pTEF-RePCT and pTEF-RePCT Yl*crc1*Δ strains on D2P0.5A1 and D3P0.5 lipogenic media. The strains were cultivated on nitrogen-limited media (1.5 g/L NH4Cl) for 120 hours at 28°C. Averages and standard errors were obtained from at least three replicate experiments. **Additional Table 4.** Primers used in this study. **Additional Figures: Additional Figure 1.** Alignment of amino acid sequences of CAC mitochondrial carriers. Alignment of CAC transporters of *H. sapiens* (HsCact), *A. nidulans* (AnCac), *S. cerevisiae* (ScCrc1) and its closest homologs in *Y. lipolytica*, YlCrc1 and YALI0A20988p. The three signature motifs of the mitochondrial carrier family of transporters, PX[DE]XX[RK]X[RK] are underlined; in black box the distinct motif RXXPANAAXF of CAC transporters and in red the identical amino acids. Sequences were aligned with NPS@: Network Protein Sequence Analysis. **Additional Figure 2.** Comparison of carbon source consumption. Comparison of carbon source consumption in wild type (blue) and strain with deletion of Yl*CRC1* (green). A) Glucose, B) acetate, and C) ethanol. For ethanol a correction based on the evaporation rate was applied. Averages and standard errors were obtained from two replicate experiments. **Additional Figure 3.** Subcellular localization of YlCrc1 mitochondrial carrier. Subcellular localization of YlCrc1 fusion protein after expression in *Y. lipolytica CRC1* knock-out strain. MitoTracker Green was used to locate mitochondria in the cells (MitoTracker panel), and phase contrast microscopy to monitor the integrity of the cells. The same cells were photographed first with a RedStar2 filter set and then with the MitoTracker filter set. Identical fields are presented. **Additional Figure 4.** Alignment of amino acid sequences of CAT enzymes of *S. cerevisiae* and *Y. lipolytica*. Multiple alignment of the five amino acid sequences of the CATs of *S. cerevisiae* and the putative CATs of *Y. lipolytica*. Cat2 and YALI0B10340p contain a mitochondrial targeting sequence (MTS) at the N-terminus (yellow) and a functional variant of the peroxisomal targeting signal type 1 (PTS-1) AKL at C-terminal (green). Cat2 and YALI0B10340p display moreover a second in-frame ATG codon, placed after the MTS (M in red). All five sequences show the LPXLPXPXL motif of choline and carnitine acetyltransferases (black box). Sequences were aligned with NPS@: Network Protein Sequence Analysis. **Additional Figure 5.** Oleate-response element (ORE). Comparison of the oleate-response element (in bold) of *S. cerevisiae* (Sc_crc1) and that of *Y. lipolytica* (Yl_crc1 and YALIA20988) found based on 5’-CGGN3TNAN9-12CCG-3’ sequence. In grey, the start of the CDS with the ATG initiation codon underlined. **Additional Figure 6.** 10.1186/s12934-023-02137-8 Real-time qPCR of mRNAs expression of Yl*CRC1*, YALI0A20988, Yl*CAT2* and Yl*YAT1* on lipogenic media. Expression of Yl*CRC1*, YALI0A20988g, Yl*CAT2* and Yl*YAT1* in wild-type cells grown on D2P.05A1 (light blue) and D3P0.5 (green) media after 15 hours (exponential phase) (A) and 72 hours (stationary phase) (B). mRNA levels were quantified by qPCR, and the mRNA of Yl*CRC1* gene, grown on glucose supplemented medium at 15 hours, was used as calibrator. The quantification of relative gene expression was calculated according to the comparative method (2-^ΔΔCCt^). Averages and standard errors were obtained from at least three independent experiments.

## Data Availability

The datasets used and/or analysed during the current study are available from the corresponding author on reasonable request.
