# Supplementary material for: Genetic inactivation of the Carnitine/Acetyl-Carnitine mitochondrial carrier of Yarrowia lipolytica leads to enhanced odd-chain fatty acid production
Source: Microb Cell Fact. 2023 Jul 13;22:128. doi: 10.1186/s12934-023-02137-8 (PMC10339547; doi:10.1186/s12934-023-02137-8)
Supplement: Supplementary file 1 — Additional Tables: Additional Table 1. Identity Matrix of CAC mitochondrial carriers. Percentage of amino acidic identity among Carnitine/Acetyl-Carnitine mitochondrial carriers of Saccharomyces cerevisiae (ScCrc1), Aspergillus nidulans (AnCac), Homo sapiens (HsCact) and the two putative CAC transporters of Yarrowia lipolytica (YlCrc1 and YALI0A20988p). The identity matrix was created by using the web tool “SIAS Sequence Identity And Similarity”. Additional Table 2. Identity Matrix of CAT enzymes of S. cerevisiae and Y. lipolytica. Percentage of amino acids identity among carnitine acetylcarnitine transferases (CATs) of S. cerevisiae (Cat2, Yat1 and Yat2) and the two putative CAT enzymes of Y. lipolytica (YALI0B10304p and YALI0F21197p). The identity matrix was created by using the web tool “SIAS Sequence Identity And Similarity”. Additional Table 3. Lipid profile of Y. lipolytica strains. Lipid profile (% of total lipids) of wild type (WT), Ylcrc1?, pTEF-RePCT and pTEF-RePCT Ylcrc1Δ strains on D2P0.5A1 and D3P0.5 lipogenic media. The strains were cultivated on nitrogen-limited media (1.5 g/L NH4Cl) for 120 hours at 28°C. Averages and standard errors were obtained from at least three replicate experiments. Additional Table 4. Primers used in this study. Additional Figures: Additional Figure 1. Alignment of amino acid sequences of CAC mitochondrial carriers. Alignment of CAC transporters of H. sapiens (HsCact), A. nidulans (AnCac), S. cerevisiae (ScCrc1) and its closest homologs in Y. lipolytica, YlCrc1 and YALI0A20988p. The three signature motifs of the mitochondrial carrier family of transporters, PX[DE]XX[RK]X[RK] are underlined; in black box the distinct motif RXXPANAAXF of CAC transporters and in red the identical amino acids. Sequences were aligned with NPS@: Network Protein Sequence Analysis. Additional Figure 2. Comparison of carbon source consumption. Comparison of carbon source consumption in wild type (blue) and strain with deletion of YlCRC1 (green). A) Gl [file 12934_2023_2137_MOESM1_ESM.docx]

**“Genetic inactivation of the Carnitine/Acetyl-carnitine mitochondrial carrier of *Yarrowia lipolytica* leads to enhanced odd-chain fatty acid production”**

Eugenia Messina, Camilla Pires de Souza, Claudia Cappella, Simona Nicole Barile, Pasquale Scarcia, Isabella Pisano, Luigi Palmieri, Jean-Marc Nicaud, Gennaro Agrimi

**Supplementary Materials**

**Additional Tables**

**Additional Table 1.** Identity Matrix of CAC mitochondrial carriers

Percentage of amino acidic identity among Carnitine/Acetyl-Carnitine mitochondrial carriers of Saccharomyces cerevisiae (ScCrc1), Aspergillus nidulans (AnCac), Homo sapiens (HsCact) and the two putative CAC transporters of Yarrowia lipolytica (YlCrc1 and YALI0A20988p). The identity matrix was created by using the web tool “SIAS Sequence Identity And Similarity” [1].

| *Percentage of identity (%)* | **YlCrc1** | **ScCrc1** | **AnCac** | **HsCact** | **YALI0A20988p** |
| --- | --- | --- | --- | --- | --- |
| **YlCrc1** | 100 | 50.00 | 59.87 | 37.87 | 26.43 |
| **ScCrc1** | 50.00 | 100 | 43.55 | 31.89 | 24.92 |
| **AnCac** | 59.87 | 43.55 | 100 | 41.52 | 26.18 |
| **HsCact** | 37.87 | 31.89 | 41.52 | 100 | 28.23 |
| **YALI0A20988p** | 26.43 | 24.92 | 26.18 | 28.23 | 100 |

**Additional Table 2.** Identity Matrix of CAT enzymes of *S. cerevisiae* and *Y. lipolytica*

Percentage of amino acids identity among carnitine acetylcarnitine transferases (CATs) of S. cerevisiae (Cat2, Yat1 and Yat2) and the two putative CAT enzymes of Y. lipolytica (YALI0B10304p and YALI0F21197p). The identity matrix was created by using the web tool “SIAS Sequence Identity And Similarity” [1].

| *Percentage of identity (%)* | **Cat2** | **Yat1** | **Yat2** | **YALI0B10304p** | **YALI0F21197p** |
| --- | --- | --- | --- | --- | --- |
| **Cat2** | 100 | 22.38 | 19.85 | 38.70 | 25.52 |
| **Yat1** | 22.38 | 100 | 29.40 | 27.58 | 48.03 |
| **Yat2** | 19.85 | 29.40 | 100 | 19.51 | 30.38 |
| **YALI0B10304p** | 38.70 | 27.58 | 19.51 | 100 | 27.90 |
| **YALI0F21197p** | 25.52 | 48.03 | 30.38 | 27.90 | 100 |

**Additional Table 3.** Lipid profile of Y. lipolytica strains

Lipid profile (% of total lipids) of wild type (WT), Ylcrc1Δ, pTEF-RePCT and pTEF-RePCT Ylcrc1Δ strains on D2P0.5A1 and D3P0.5 lipogenic media. The strains were cultivated on nitrogen-limited media (1.5 g/L NH_4_Cl) for 120 hours at 28°C. Averages and standard errors were obtained from at least three replicate experiments.

|  | **FAs content (%) D2P0.5A1** | | | | | | | | | | | | |
| --- | --- | --- | --- | --- | --- | --- | --- | --- | --- | --- | --- | --- | --- |
|  | C14:0 | C15:0 | C16:0 | C16:1 | C17:0 | C17:1 | C18:0 | C18:1 | C18:2 | C19:0 | C20:0 | C21:0 | C23:0 |
| WT | 0.02 ± 0.02 | 0.66 ± 0.21 | 11.39 ± 0.23 | 4.49 ± 1.45 | 1.42 ± 0.28 | 7.03 ± 0.77 | 10.11 ± 0.56 | 49.13 ± 2.05 | 14.35 ± 1.64 | 0.35 ± 0.18 | 0.36 ± 0.12 | 0.18 ± 0.09 | 0.01 ± 0.01 |
| Ylcrc1Δ | 0.53 ± 0.47 | 1.42 ± 1.01 | 14.29 ± 1.09 | 3.34 ± 1.54 | 2.03 ± 0.37 | 6.87 ± 1.96 | 12.37 ± 2.12 | 41.1 ± 3.98 | 16.24 ± 2.54 | 0.15 ± 0.11 | 0.31 ± 0.09 | 0.13 ± 0.07 | 0.04 ± 0.02 |
| pTEF-RePCT | 0.02 ± 0.01 | 2.28 ± 0.46 | 4.42 ± 0.67 | 0.89 ± 0.44 | 7.86 ± 1.03 | 53.8 ± 1.71 | 2.99 ± 0.46 | 19.63 ± 1.55 | 5.42 ± 0.34 | 0.83 ± 0.07 | 0.04 ± 0.02 | 0.25 ± 0.09 | 0.38 ± 0.06 |
| pTEF-RePCT Ylcrc1Δ | 0.06 ± 0.03 | 0.94 ± 0.10 | 10.21 ± 0.54 | 2.89 ± 0.54 | 4.67 ± 0.30 | 18.63 ± 1.63 | 9.93 ± 0.79 | 38.27 ± 1.06 | 12.12 ± 1.21 | 0.62 ± 0.21 | 0.34 ± 0.13 | 0.44 ± 0.22 | 0.29 ± 0.16 |

|  | **FAs content (%) D3P0.5** | | | | | | | | | | | | |
| --- | --- | --- | --- | --- | --- | --- | --- | --- | --- | --- | --- | --- | --- |
|  | C14:0 | C15:0 | C16:0 | C16:1 | C17:0 | C17:1 | C18:0 | C18:1 | C18:2 | C19:0 | C20:0 | C21:0 | C23:0 |
| WT | 0.46 ± 0.43 | 0.71 ± 0.46 | 13.66 ± 1.31 | 6.70 ± 0.54 | 1.67 ± 0.38 | 12.96 ± 1.35 | 6.48 ± 0.76 | 42.71 ± 1.07 | 12.85 ± 0.88 | 0.38 ± 0.19 | 0.22 ± 0.14 | 1.20 ± 0.82 | 0.0  ±  0.0 |
| Ylcrc1Δ | 0.04 ± 0.01 | 0.31 ± 0.08 | 18.15 ± 0.43 | 5.41 ± 0.95 | 0.77 ± 0.12 | 9.11 ± 0.41 | 6.27 ± 0.78 | 44.79 ± 3.35 | 14.82 ± 0.51 | 0.11 ± 0.02 | 0.11 ± 0.14 | 0.07 ± 0.01 | 0.05 ± 0.01 |
| pTEF-RePCT | 0.07 ± 0.04 | 6.37 ± 2.15 | 5.15 ± 2.16 | 0.25 ± 0.14 | 4.67 ± 1.03 | 64.33 ± 6.03 | 0.88 ± 0.45 | 12.11 ± 1.71 | 5.49 ± 1.60 | 0.34 ± 0.18 | 0.0  ±  0.0 | 0.16 ± 0.08 | 0.19 ± 0.11 |
| pTEF-RePCT Ylcrc1Δ | 0.07 ± 0.03 | 0.81 ± 0.35 | 12.83 ± 1.94 | 4.15 ± 1.09 | 3.11 ± 0.89 | 16.92 ± 0.63 | 10.0  ± 1.87 | 42.26 ± 1.92 | 8.01 ± 1.73 | 0.53 ± 0.33 | 0.48 ± 0.28 | 0.40 ± 0.23 | 0.44 ± 0.32 |

**Additional Table 4.** Primers used in this study.

| **Name** | **Sequence (5’->3’)** | **Purpose** |
| --- | --- | --- |
| 02431gRNAFw2 | TTCGATTCCGGGTCGGCGCAGGTTGGGTGTAGCGGCGGGTTTAGGGTTTTA | Forward gRNA outside the CDS of Yl*CRC1* |
| 02431gRNARv2 | GCTCTAAAACCCTAAACCCGCCGCTACACCCAACCTGCGCCGACCCGGAAT | Reverse gRNA outside the CDS of Yl*CRC1* |
| 02431gRNAFw4 | TTCGATTCCGGGTCGGCGCAGGTTGATGGGGGTGACTCCGACCAGGTTTTA | Forward gRNA inside the CDS of Yl*CRC1* |
| 02431gRNARv4 | GCTCTAAAACCTGGTCGGAGTCACCCCCATCAACCTGCGCCGACCCGGAAT | Reverse gRNA inside the CDS of Yl*CRC1* |
| VerifsgRNA_Fw | CTTTGAAAAATACCTCTAATGCGCC | PCR verification of paired gRNAs insertion in JME4390 and JME4472 plasmids |
| VerifsgRNA_Rv | AAGCACCGACTCGGTGCCA |  |
| pSCR1_intern_Fw | CGGACACTTCTTCGTGTATGAGAC | Sequencing of plasmids JME5627 and JME5628 |
| ARS1_intern_Rev | GGTGATTTGGATCTAAGGTTCGTAC |  |
| C02431VER1synt | CTTGTATCTTAGTGCCTCCGA | PCR verification of Yl*CRC1* deletion |
| C02431REV4synt | GTCATCATCTCGTCTGTCCG |  |
| C02431VER2synt | CGTCTGGTCTGGAGCTATAT | Sequencing of *Y. lipolytica* genome for verification of Yl*CRC1* deletion |
| C02431BamHIFw | GGGGGATCCCACAATGAGTGACGCTCCCGAAATTGA | Amplification of *Y. lipolytica* genome for the construction of plasmids JME5646 and p-M4B361 |
| C02431AvrIIRv | CCCCCTAGGCTAAGCGAGAGAAGTGAAGAACTTGTGGG | Amplification of *Y. lipolytica* genome for the construction of plasmid JME5646 |
| P_TEF_Intern_Fw | TCTGGAATCTACGCTTGTTCAG | Sequencing of plasmid JME5646 |
| ZetaDown_Intern-Rv | GGTAACGCCGATTCTCTCTG |  |
| URA3M_intern_Fw | CATCCAGAGAAGCACACAGG | PCR verification of the insertion of Yl*CRC1* overexpression cassette |
| C02431_internal_Rev | AGGAACTCGTAGGTAGCAAAG |  |
| C02431_internal_Fw | ACAAGGAGGGTGGTCTCAAG |  |
| T_Lip2_Intern-Rv | GATTTGTCTTAGAGGAACGCATA |  |
| GATC-TEFStart Fw | GGGTATAAAAGACCACCGTCC | PCR verification of the insertion of RePCT overexpression cassette |
| RePCT-internal-Rev | CCAATGGGGCCTGCCTC |  |
| CRC1_AvrII_noSTOP | CCCCCTAGGGCAGCGAGAGAAGTGAAGAACTTGTG | Amplification of *Y. lipolytica* genome for the construction of plasmid p-M4B361 |
|  |  |  |
| CRC1 Forward | CGTGGTCGGTAGCATCATGA | qPCR of Yl*CRC1* |
| CRC1 Reverse | AGCCATGACTCCTCGGTAGAAG |  |
| CRC1b Forward | GCAGCTCCCATTGAACACATC | qPCR of YALI0A20988g |
| CRC1b Reverse | TGGTCTTTGCGTCGTATTGG |  |
| CAT2 Forward | CTTCTGCTGGTAAGGGTGTTGAC | qPCR of YALI0B10340g |
| CAT2 Reverse | GCCCTCCTTGAGGCACTTC |  |
| YAT1 Forward | AGCTGCCCCCAGATAACGT | qPCR of YALI0F21197g |
| YAT1 Reverse | TCGCGAGTACTGGTACATGCA |  |
| TPI1 Forward | CAAGACCGAGGTGGTCATCTC | qPCR of Yl*TPI1* |
| TPI1 Reverse | AGACACGGTGGGCTTCTTCA |  |

**Additional Figures**

**Additional Figure 1.** Alignment of amino acid sequences of CAC mitochondrial carriers

Alignment of CAC transporters of *H. sapiens* (HsCact), *A. nidulans* (AnCac), *S. cerevisiae* (ScCrc1) and its closest homologs in *Y. lipolytica*, YlCrc1 and YALI0A20988p. The three signature motifs of the mitochondrial carrier family of transporters, PX[DE]XX[RK]X[RK] are underlined; in black box the distinct motif RXXPANAAXF of CAC transporters [2] and in red the identical amino acids. Sequences were aligned with NPS@: Network Protein Sequence Analysis [3].

**
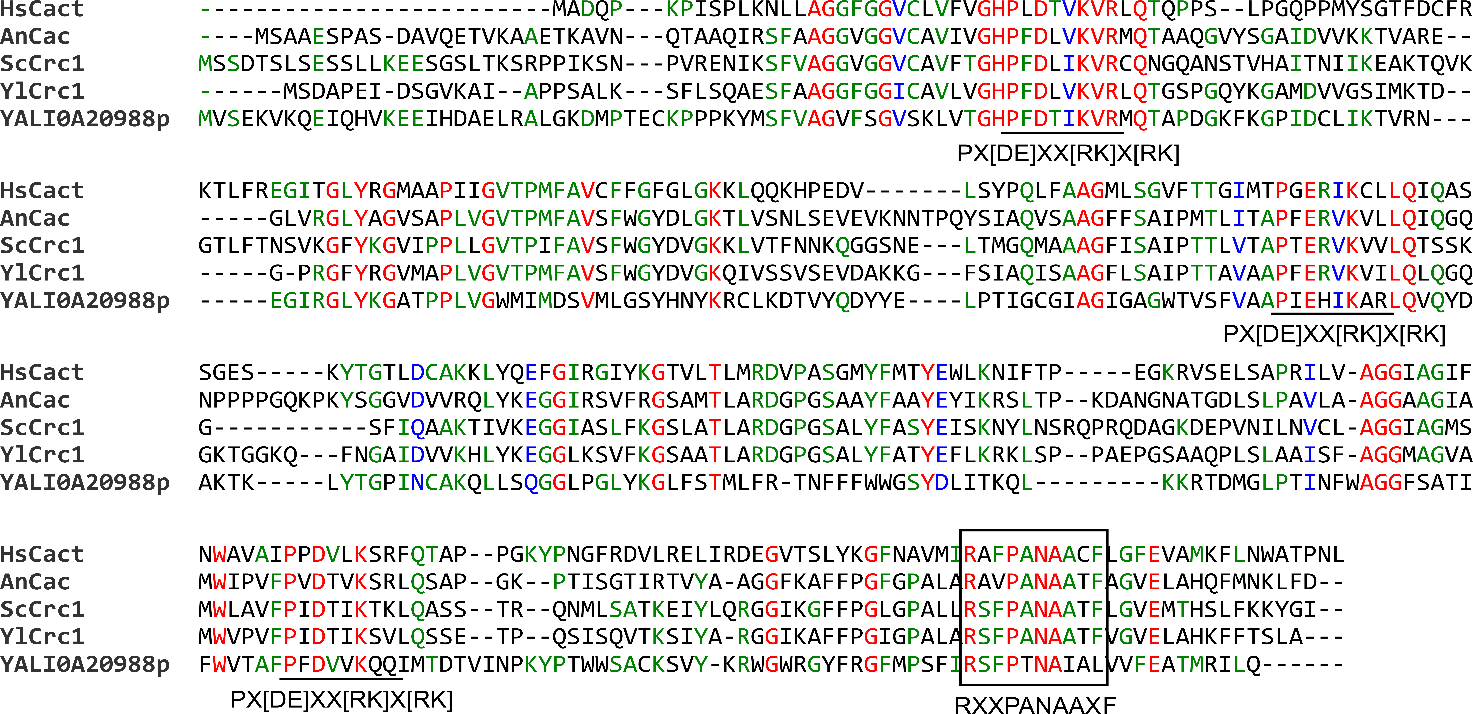
**

**Additional Figure 2.** Comparison of carbon source consumption.

Comparison of carbon source consumption in wild type (blue) and strain with deletion of Yl*CRC1* (green). **A**) Glucose, **B**) acetate, and **C**) ethanol. For ethanol a correction based on the evaporation rate was applied. Averages and standard errors were obtained from two replicate experiments.


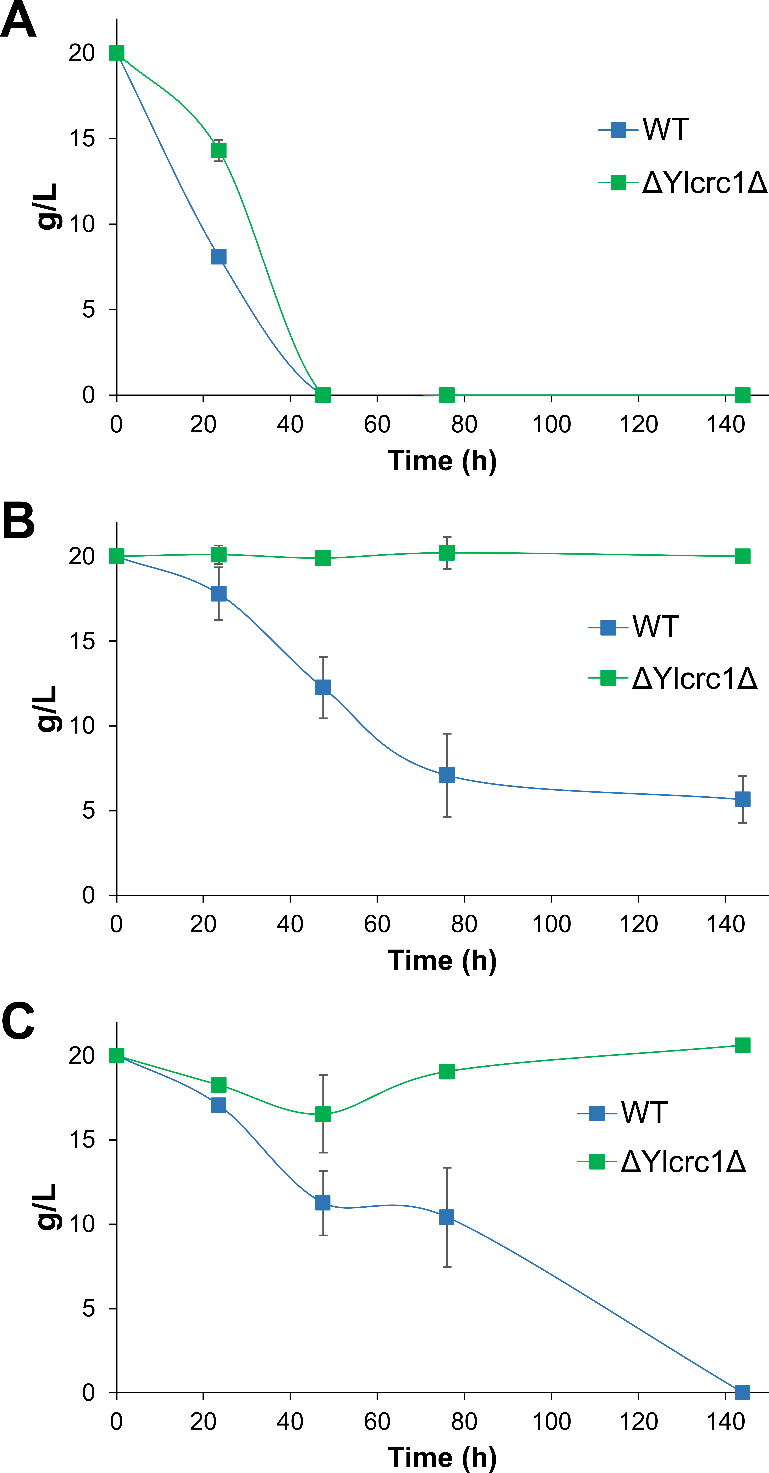


**Additional Figure 3.** Subcellular localization of YlCrc1 mitochondrial carrier

Subcellular localization of YlCrc1 fusion protein after expression in *Y. lipolytica* *CRC1* knock-out strain. MitoTracker Green was used to locate mitochondria in the cells (MitoTracker panel), and phase contrast microscopy to monitor the integrity of the cells. The same cells were photographed first with a RedStar2 filter set and then with the MitoTracker filter set. Identical fields are presented.


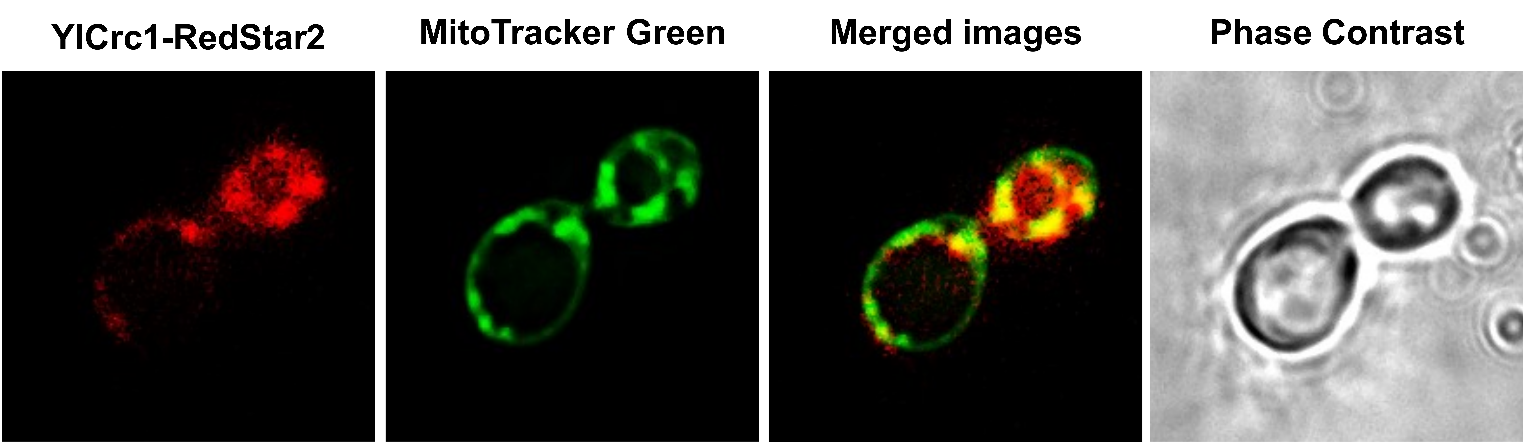


**Additional Figure 4.** Alignment of amino acid sequences of CAT enzymes of *S. cerevisiae* and *Y. lipolytica*

Multiple alignment of the five amino acid sequences of the CATs of *S. cerevisiae* and the putative CATs of *Y. lipolytica*. Cat2 and YALI0B10340p contain a mitochondrial targeting sequence (MTS) at the N-terminus (yellow) and a functional variant of the peroxisomal targeting signal type 1 (PTS-1) AKL at C-terminal (green). Cat2 and YALI0B10340p display moreover a second in-frame ATG codon, placed after the MTS (M in red). All five sequences show the LPXLPXPXL motif of choline and carnitine acetyltransferases [4,5] (black box). Sequences were aligned with NPS@: Network Protein Sequence Analysis [3].


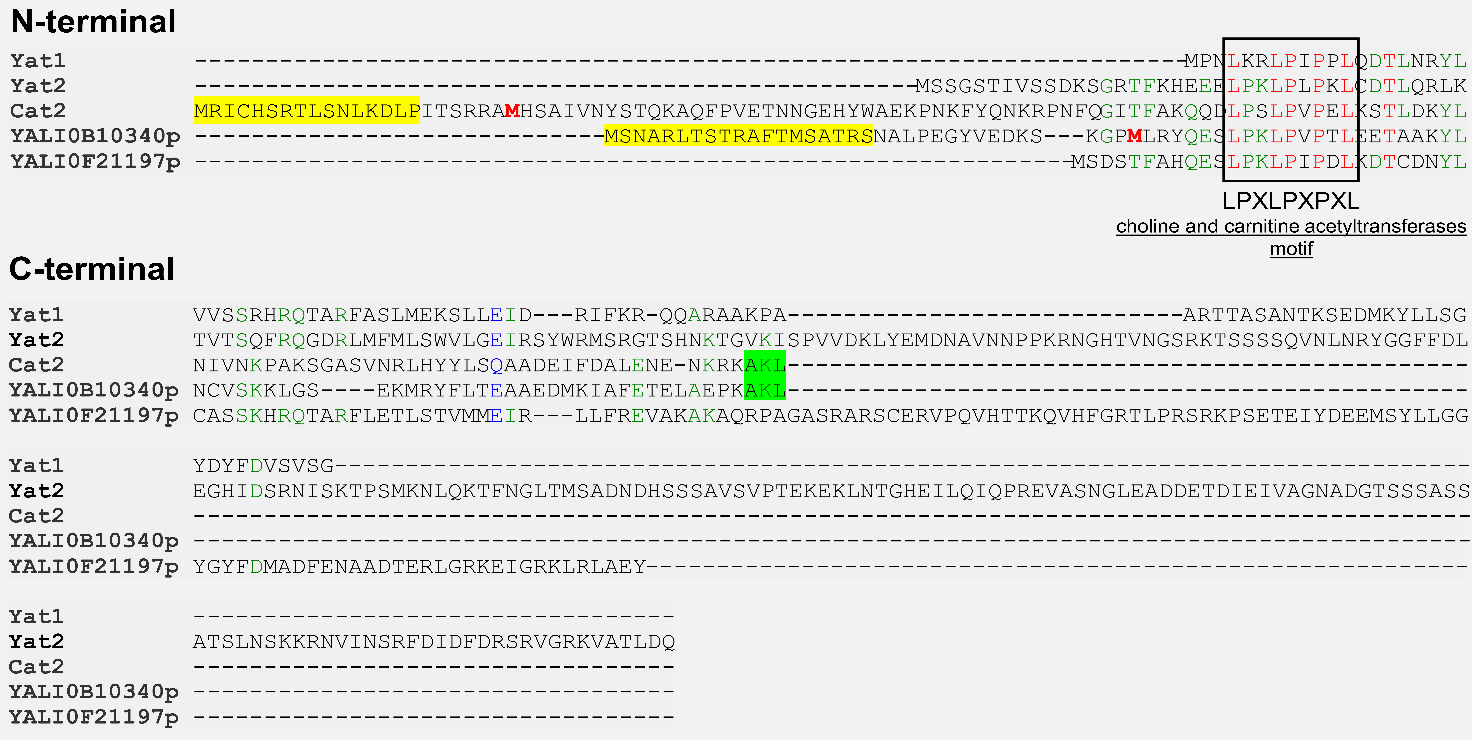


**Additional Figure 5.** Oleate-response element (ORE)

Comparison of the oleate-response element (in bold) of *S. cerevisiae* (Sc_crc1) and that of *Y. lipolytica* (Yl_crc1 and YALIA20988) found based on 5′-CGGN_3_TNAN_9–12_CCG-3′ sequence [6]. In grey, the start of the CDS with the ATG initiation codon underlined.


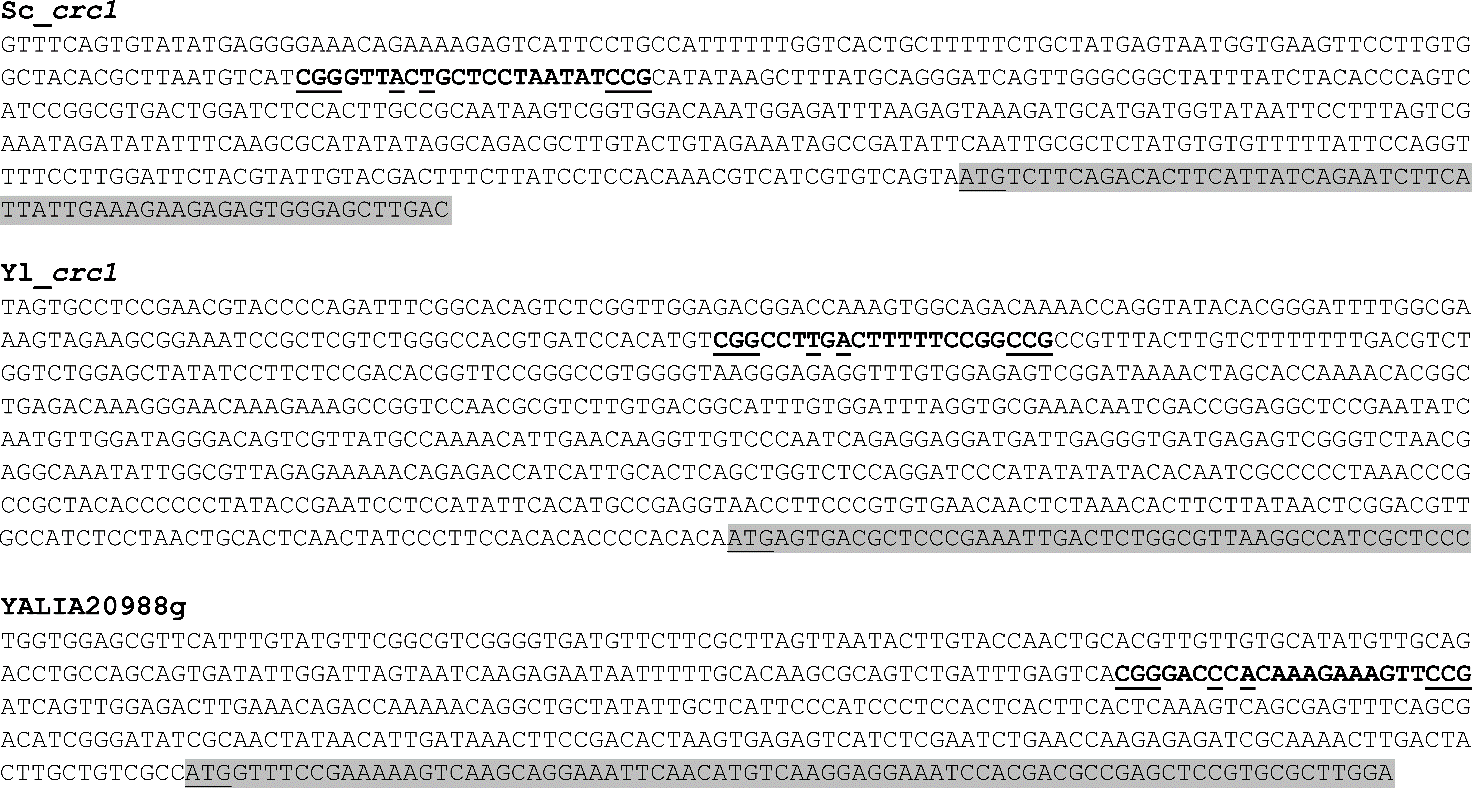


**Additional Figure 6.** Real-time qPCR of mRNAs expression of Yl*CRC1*, YALI0A20988, Yl*CAT2* and Yl*YAT1* on lipogenic media

Expression of Yl*CRC1*, YALI0A20988g, Yl*CAT2* and Yl*YAT1* in wild-type cells grown on D2P.05A1 (light blue) and D3P0.5 (green) media after 15 hours (exponential phase) (**A**) and 72 hours (stationary phase) (**B**). mRNA levels were quantified by qPCR, and the mRNA of Yl*CRC1* gene, grown on glucose supplemented medium at 15 hours, was used as calibrator. The quantification of relative gene expression was calculated according to the comparative method (2^−ΔΔCt^) [7,8]. Averages and standard errors were obtained from at least three independent experiments.


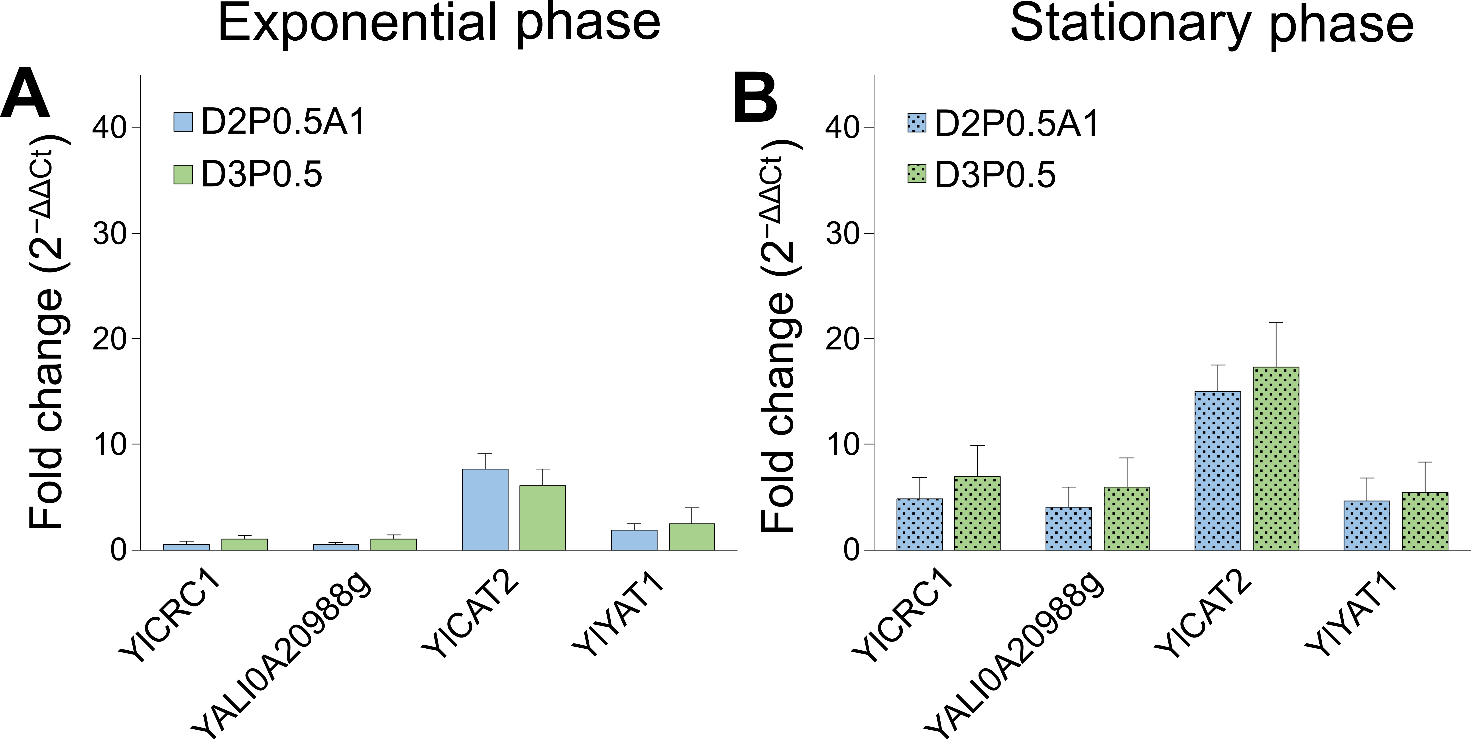


**References**

[1] Reche P. (2008) SIAS: Sequence identities and similarities. Available at: http://imed.med.ucm.es/Tools/sias.html.

[2] De Lucas JR, Indiveri C, Tonazzi A, Perez P, Giangregorio N, Iacobazzi V, Palmieri F. Functional characterization of residues within the carnitine/acylcarnitine translocase RX2PANAAXF distinct motif. Mol Membr Biol. 2008;25(2):152-63.

[3] Combet C, Blanchet C, Geourjon C, Deléage G. NPS@: network protein sequence analysis. Trends Biochem Sci. 2000;25(3):147-150.

[4] Finocchiaro G, Taroni F, Rocchi M, Martin AL, Colombo I, Tarelli GT, DiDonato S. cDNA cloning, sequence analysis, and chromosomal localization of the gene for human carnitine palmitoyltransferase. Proc Natl Acad Sci USA. 1991;15;88(2):661-5.

[5] Kispal G, Sumegi B, Dietmeier K, Bock I, Gajdos G, Tomcsanyi T, Sandor A. Cloning and sequencing of a cDNA encoding *Saccharomyces cerevisiae* carnitine acetyltransferase. Use of the cDNA in gene disruption studies. J Biol Chem. 1993; 25;268(3):1824-9.

[6] Gurvitz A, Rottensteiner H. The biochemistry of oleate induction: transcriptional upregulation and peroxisome proliferation. Biochim Biophys Acta. 2006;1763(12):1392-1402.

[7] Bustin SA. Absolute quantification of mRNA using real-time reverse transcription polymerase chain reaction assays. J Mol Endocrinol. 2000;25(2):169-193.

[8] Scarcia P, Agrimi G, Germinario L, Ibrahim A, Rottensteiner H, Palmieri F, Palmieri L. In *Saccharomyces cerevisiae* grown in synthetic minimal medium supplemented with non-fermentable carbon sources glutamate is synthesized within mitochondria. Rend. Fis. Acc. Lincei 2018;29, 483–490.
